# Emerging Wearable Acoustic Sensing Technologies

**DOI:** 10.1002/advs.202408653

**Published:** 2025-01-03

**Authors:** Tao Liu, Yuchen Mao, Hanjie Dou, Wangyang Zhang, Jiaqian Yang, Pengfan Wu, Dongxiao Li, Xiaojing Mu

**Affiliations:** ^1^ Key Laboratory of Optoelectronic Technology & Systems of Ministry of Education International R&D Center of Micro‐Nano Systems and New Materials Technology Chongqing University Chongqing 400044 China

**Keywords:** acoustic sensor, human‐machine interface, ultrasonic healthcare, wearable and implantable

## Abstract

Sound signals not only serve as the primary communication medium but also find application in fields such as medical diagnosis and fault detection. With public healthcare resources increasingly under pressure, and challenges faced by disabled individuals on a daily basis, solutions that facilitate low‐cost private healthcare hold considerable promise. Acoustic methods have been widely studied because of their lower technical complexity compared to other medical solutions, as well as the high safety threshold of the human body to acoustic energy. Furthermore, with the recent development of artificial intelligence technology applied to speech recognition, speech recognition devices, and systems capable of assisting disabled individuals in interacting with scenes are constantly being updated. This review meticulously summarizes the sensing mechanisms, materials, structural design, and multidisciplinary applications of wearable acoustic devices applied to human health and human–computer interaction. Further, the advantages and disadvantages of the different approaches used in flexible acoustic devices in various fields are examined. Finally, the current challenges and a roadmap for future research are analyzed based on existing research progress to achieve more comprehensive and personalized healthcare.

## Graphical abstract

This review summarizes recent advancements in wearable acoustic devices developed through interdisciplinary and bionic approaches. These applications include bio‐acoustic detection, ultrasound energy supply, and ultrasonic diagnosis. It highlights challenges and future directions, aiming to promote their use in the human body and explore integration opportunities with other technologies.

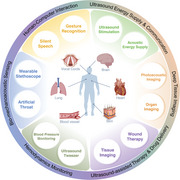

## 1 Introduction

Recent years have seen a sharp increase in the demand for healthcare resources worldwide, mainly owing to increasing aging problems, rising healthcare costs, and expansion of healthcare facilities. This, in turn, has contributed to an increasing demand for medical imaging. Most patients rely on specialized devices to obtain physiological information, which serves as an important basis for diagnosis; consequently, the use of imaging equipment is frequent and resource‐constrained. In addition, while many diseases cause minor physical abnormalities in the early stages, they can only be detected using specialized medical equipment and by a physician's diagnosis.^[^ ^1^, ^2^, ^3^ ^]^ In this context, low‐cost private medical monitoring solutions have become an effective means of alleviating the shortage of healthcare resources, creating considerable market opportunities for home‐based medical imaging equipment. In addition to imaging devices, the design of wearable devices for the functional compensation of disabled individuals is an important direction in the field of personal healthcare. Language is the primary means of communication for humans. Deaf patients, who have lost the ability to speak or hear because of congenital defects or acquired injuries, need to use sign language and hearing aids for effective communication. Therefore, developing acoustic perception and speech devices with alternative functions is important for the rehabilitation of these populations.

The emergence and flourishing of wearable sensor devices have changed the diagnostic methods in the healthcare industry, facilitating the creation of a sensor network that covers most of the human body. A personalized healthcare platform with higher levels of integration and accuracy can simultaneously provide real‐time monitoring of different organs, tissues, and biosignals (e.g., temperature,^[^ ^4^, ^5^ ^]^ blood pressure,^[^ ^6^, ^7^ ^]^ sweat^[^ ^8^ ^]^ and heart rate)^[^ ^9^ ^]^ without the need for professional operators. Long‐term monitoring and full‐dimensional bioinformation acquisition can support an automatic diagnostic artificial intelligence (AI) system to provide accurate clinical suggestions to doctors; this would reduce the analysis cost and enable real‐time adjustments to treatment strategies.^[^ ^10^, ^11^, ^12^, ^13^ ^]^ Additionally, wearable devices can be used as input devices for human–machine interfaces^[^ ^14^, ^15^, ^16^, ^17^ ^]^ By converting motion and voice signals into digital control commands, wearable devices can also convert voice signals into digital control commands.

Conventional acoustic technologies have significant limitations in the fields of medicine and health monitoring. First, these technologies typically rely on large, stationary devices, such as ultrasound imagers and stethoscopes; this limits their use in mobile and telemedicine. Second, maintenance of conventional acoustic equipment tends to be expensive; therefore, they are unsuitable for widespread use. The limited resolution and sensitivity of this equipment hinder their capability to detect early‐stage diseases or subtle physiological changes. They usually require specialized operators and have a high usage threshold, making them unsuitable for daily health monitoring at home or by individuals.

To overcome the limitations of traditional acoustic technology, researchers have explored the potential of flexible devices by employing several novel materials and processes.^[^ ^18^, ^19^, ^20^, ^21^ ^]^ Many highly stretchable, lightweight materials have been applied to the sensors, including materials with better electromechanical coupling properties and composites that can be combined in various configurations. These innovations enable wearable sensors to perfectly fit and adapt to the deformations of the body, while achieving the same detection capabilities as traditional sensors. These also present new development opportunities for rapidly expanding fields such as the Internet of Things and artificial intelligence.

**Figure 1:**
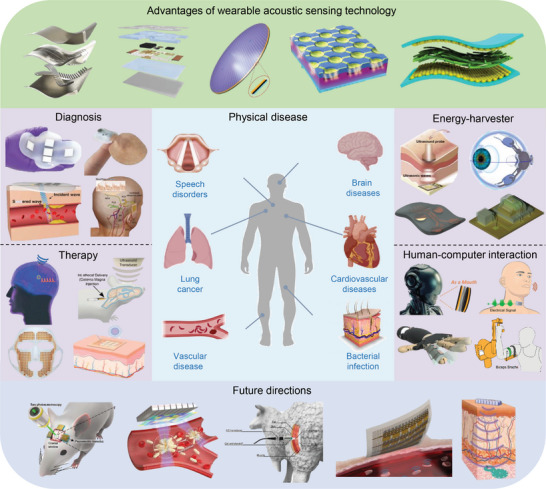
Application of flexible wearable acoustic sensors targeting different parts of the human body. (Reproduced with permission.^[^ ^22^ ^]^ Copyright 2023, Springer Nature. Reproduced with permission.^[^ ^23^ ^]^ Copyright 2016, American Association for the Advancement of Science. Reproduced with permission.^[^ ^24^ ^]^ Copyright 2015, Wiley. Reproduced with permission.^[^ ^25^ ^]^ Copyright 2020, Springer Nature. Reproduced with permission.^[^ ^26^ ^]^ Copyright 2019, American Chemical Society. Reproduced with permission.^[^ ^27^ ^]^ Copyright 2024, Springer Nature. Reproduced with permission.^[^ ^28^ ^]^ Copyright 2022, American Association for the Advancement of Science. Reproduced with permission.^[^ ^29^ ^]^ Copyright 2021, American Association for the Advancement of Science. Reproduced with permission.^[^ ^30^ ^]^ Copyright 2023, Springer Nature. Reproduced with permission.^[^ ^31^ ^]^ Copyright 2022, Elsevier. Reproduced with permission.^[^ ^32^ ^]^ Copyright 2024, Springer Nature. Reproduced with permission.^[^ ^33^ ^]^ Copyright 2023, Wiley. Reproduced with permission.^[^ ^34^ ^]^ Copyright 2021, Springer Nature. Reproduced with permission.^[^ ^35^ ^]^ Copyright 2022, American Association for the Advancement of Science. Reproduced with permission.^[^ ^36^ ^]^ Copyright 2021, Wiley. Reproduced with permission.^[^ ^37^ ^]^ Copyright 2022, American Chemical Society. Reproduced with permission.^[^ ^38^ ^]^ Copyright 2022, Elsevier. Reproduced with permission.^[^ ^39^ ^]^ Copyright 2023, Wiley. Reproduced with permission.^[^ ^40^ ^]^ Copyright 2024, Wiley. Reproduced with permission.^[^ ^41^ ^]^ Copyright 2024, Springer Nature. Reproduced with permission.^[^ ^42^ ^]^ Copyright 2019, American Chemical Society. Reproduced with permission.^[^ ^43^ ^]^ Copyright 2023, Springer Nature. Reproduced with permission.^[^ ^44^ ^]^ Copyright 2023, Springer Nature. Reproduced with permission.^[^ ^45^ ^]^ Copyright 2022, Springer Nature. Reproduced with permission.^[^ ^46^ ^]^ Copyright 2024, American Association for the Advancement of Science.

This review focuses on a variety of wearable acoustic sensors and actuators applied to the human body and their applications in healthcare, biology, and human–machine interfaces (**Figure** **1**). Section 2 describes the sensing principles of the presented acoustic devices and the advantages of these principles in the development of wearable devices, including the piezoelectric, piezoresistive, capacitive, and triboelectric principles. Section 3 focuses on wearable devices for bioacoustic signal detection, which are sensors used to obtain physiological acoustic signals in the human body, in addition to bionic wearable acoustic sensors inspired by the physiological structures of the human body or other living organisms. Section 4 introduces wearable or implantable acoustic devices that provide in vivo energy supply, communication, and therapeutic functions through ultrasound, alongside the treatment of the human body through ultrasound. Section 5 details wearable imagers that target different parts of the human body using different methods and evaluates the imaging effect of these devices. Section 6 summarizes several cutting‐edge forms of ultrasonic applications in recent years and discusses the possibility of combining them into wearable devices. Finally, Section 7 discusses the development of wearable acoustic devices in human health and human–machine interfaces (HMIs), as well as future directions and possible challenges.

## 2 Working Mechanism and Architecture of Acoustic Devices

### 2.1 Piezoelectric Mechanism

Piezoelectric acoustic transducers are preferred for the development of wearable acoustic devices owing to their fast response and high mechanical‐to‐electrical energy conversion efficiency. The piezoelectric equation describes the relationship between the electrical and mechanical changes^[^ ^47^ ^]^

(1) $$\left[\begin{array}{c} D\\ S \end{array}\right]=\left[\begin{array}{cc} \varepsilon^{T} & d\\ d & s^{E} \end{array}\right]\cdot \left[\begin{array}{c} E\\ T \end{array}\right]$$

where *D* and S represent the potential shift and strain of the piezoelectric material, respectively; T denotes the stress tensor; and *E* is the electric field vector. *d* is the piezoelectric constant, which represents the change in the strain component owing to a change in the strength of the unit electric field at constant stress. ε^*T*^is the dielectric constant obtained at constant stress, called the accessible dielectric constant. *s^E^* is the smoothness elasticity coefficient obtained at a constant electric field and is called the short‐circuit smoothness elasticity coefficient.

**Figure 2:**
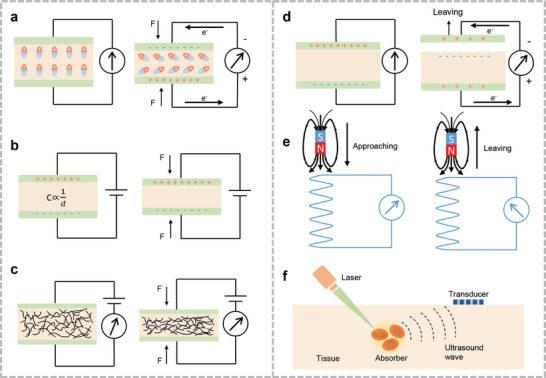
Different kinds of acoustic sensing mechanisms: a) Piezoelectric sensing mechanism. b) Capacitive sensing mechanism. c) Piezoresistive sensing mechanism. d) Triboelectric sensing mechanism. e) Electromagnetic sensing mechanism. f) Photoacoustic sensing mechanism.

The positive and negative piezoelectric effects of the piezoelectric materials were used for acoustic sensing and ultrasound emission, respectively (**Figure** **2**a). The relationship between the mechanical direction and polarization direction can be classified into two modes of operation, *d_31_* and *d_33_*, which are orthogonal or parallel to the mechanical direction. Acoustic devices based on the piezoelectric effect include microphones/speakers, ultrasonic transducers, sonobuoys, and piezoelectric vibration energy harvesters. These acoustic devices utilize piezoelectric ceramics (including barium titanate (BTO),^[^ ^48^ ^]^ lead zirconate titanate (PZT),^[^ ^49^ ^]^ and lead meta‐niobate (PN)), piezoelectric single crystals (including lithium niobate oxide (LiNbO_3_),^[^ ^50^ ^]^ (NaNbO_3_),^[^ ^51^ ^]^ etc.), and piezoelectric polymers (e.g., polyvinylidene fluoride (PVDF),^[^ ^52^ ^]^ and poly(vinylidene fluoride) copolymer (P(VDF‐TrFE)))^[^ ^53^ ^]^ as active cores to convert mechanical vibration and electrical signal into each other. Block piezoelectric ceramics are widely used in medical ultrasound probes and industrial nondestructive testing (NDT) because of their low processing cost and electromechanical conversion efficiency. However, their large size and high driving power have prevented their widespread use in flexible acoustic devices. Commonly used piezoelectric materials, such as PZT and Pb(Mg_1/3_Nb_2/3_)O_3_‐PbTiO_3_ (PMN‐PT), contain lead, which poses a threat to human health. Piezoelectric polymers have become the primary choice for flexible wearable acoustic devices owing to their biocompatibility, flexibility, and plasticity. However, their low electromechanical coupling coefficients (typically <1%) and susceptibility to temperature variations require careful consideration during design.

### 2.2 Capacitive Mechanism

Capacitive acoustic devices convert acoustic vibrations into electrical signals by converting them into changes in the distances between capacitive parallel electrodes. The conversion was performed using the following formula:^[^ ^54^, ^55^, ^56^, ^57^, ^58^ ^]^

(2) $$C=\frac{\varepsilon_{0}\varepsilon_{r}S}{d}$$

where ε_0_ and ε_*r*_ represent the vacuum dielectric constant and the relative dielectric constant of the medium, respectively. Further, *S* represents the overlapping area between the upper and lower electrode plates; and *d* denotes the distance between the two electrodes. A common condenser acoustic microphone consists of two parallel plates: a movable top diaphragm and a fixed back plate (Figure 2b). An air gap separates them and an insulator is used as the spacer. The top and bottom plates are connected to electrodes to measure the output signal. When acoustic pressure is applied to the diaphragm, it vibrates, causing the air gap to change. Compared with piezoelectric materials, sensors with capacitive structures offer better temperature tolerance and adequate frequency responses.^[^ ^59^ ^]^ Capacitive acoustic sensors with an extremely small air gap developed through the microelectromechanical system (MEMS) process dramatically improve the auditory sensitivity and low‐acoustic pressure sensing. Their bandwidths cover the frequency range of human vocalizations. The sensitivity can also be optimized by filling different dielectric materials between the diaphragms to adjust ε_*r*_. In addition, when the capacitor is worn on the body, the sensor can obtain the maximum intensity of the acoustic signal and the ambient noise, which is insulated by the human skin, considerably reducing the interference level. However, because they need to be worn on the human body, the flexibility and biocompatibility of the material selection need to be prioritized. Moreover, the packaging of the device must be resistant to corrosion due to perspiration so that the performance of the device is not affected.

### 2.3 Piezoresistive Mechanism

Piezoresistive acoustic sensors convert acoustic–mechanical vibrations into changes in electrical resistance through the piezoresistive effect (Figure 2c). Piezoresistivity is produced by the deformation of energy bands due to applied stress, and the deformed energy bands affect the mobility of electrons and holes, thus changing the resistivity.^[^ ^60^ ^]^ It is common to measure the piezoresistivity of a material using the gauge factor, which is defined as the ratio of the rate of resistance change to the mechanical strain due to pressure:^[^ ^61^, ^62^ ^]^

(3) $$GF=\frac{\Delta R/R}{\varepsilon }=\frac{\Delta \rho /\rho }{\varepsilon }+\left(1+2\gamma \right)$$

where *R*, ρ, and γ represent material resistance, resistivity, and Poisson's ratio, respectively. Metals and semiconductors are the most commonly used piezoresistive materials. The length and cross‐sectional area of metallic materials change when subjected to an external pressure, which alters their resistance; however, the changes in their resistivity are small. Semiconductor materials exhibit limited mechanical deformations. When subjected to an external force, the internal lattice is distorted, altering the paths of electrons and holes and thus affecting the resistivity. Both materials have advantages in terms of manufacturing costs; however, their weaker conformability and tensile properties make them difficult to apply directly in wearable scenarios.^[^ ^62^ ^]^

To overcome the inherent shortcomings of metals and semiconductors, many researchers have recently used conducting polymers as piezoresistive sensitive elements.^[^ ^26^, ^63^, ^64^, ^65^ ^]^ The piezoresistive properties of conducting polymer materials are achieved through a variety of mechanisms such as internal conductive networks, tunnel effects, structural changes in the polymer chains, and interfacial effects. Conductive polymers commonly used in piezoresistive sensors include polypyrrole, polyaniline (PANI), and poly(3,4‐ethylene dioxythiophene): poly(styrene sulfonate) (PEDOT:PSS). Their main advantages include adjustable conductivity and excellent flexibility; however, high temperatures, humidity, chemical corrosion, and other factors weaken their stability.^[^ ^62^ ^]^ Conductive polymers can be used as conductive fillers, and nonconductive polymers can be used to form new composites and overcome their defects. By adjusting the filler proportion, the piezoresistive properties can be tailored to meet the requirements. The change in the resistance of the composite material depends on the elastic deformation of the substrate material under force. A rigid substrate makes it difficult for a composite to respond to small forces. A soft substrate affects the overall stability and reduces sensing sensitivity. In contrast to the filling method, when subjected to an external pressure, the conductive paths between the layers are partially broken and reconnected after the disappearance of the external force, resulting in a corresponding change in the resistance and further broadening the potential of piezoresistive applications.^[^ ^66^, ^67^ ^]^

### 2.4 Triboelectric Mechanism

Frictional charging is one of the most common physical phenomena in daily life and arises from charge transfer between different materials when they are separated by contact (Figure 2d). In 2012, Wang et al. proposed a triboelectric nanogenerator (TENG).^[^ ^68^ ^]^ The principle is that the tribo‐pair material between two polar plates generates a transfer charge after contact, and the overall capacitance changes owing to the polarized triboelectric charge and the change in the spacing between the two polar plates at the time of separation, forming a voltage difference between the two electrodes, which can be expressed as follows:^[^ ^69^, ^70^ ^]^

(4) $$U=U_{oc}\left(d\right)-\frac{Q}{C(d)}$$

where *U_OC_* represents the open‐circuit power due to the polarization charge, *Q* represents the charge transferred between the two electrodes, *C* represents the capacitance formed between the two electrode plates, and both *U_OC_* and *C* are functions of the spacing between the electrode plates. This equation is the governing equation for any TENG, and clearly explains its inherent capacitive behavior. The polarity of the tribe pair material directly determines the amount of charge transferred during contact separation. The larger the difference in polarity between the two materials, the larger the amount of charge exchanged.

For different acoustic frequency bands, TENG can be divided into two categories: the detection of low‐ and medium‐frequency audible acoustic signals, and the collection of high‐frequency ultrasonic acoustic energy. With its unique working mechanism, the acoustic sensing transducer based on the TENG not only demonstrates ultra‐high audible sensitivity and an extremely wide frequency band but also reduces energy consumption and increases the service life of the device owing to its self‐supply characteristics. In contrast to piezoelectric energy collection, TENG can be selected from a wide range of flexible organic materials with good biocompatibility and high charge conversion efficiency. It also has a relatively simple structural design and low manufacturing cost, which are conducive to large‐scale manufacturing.^[^ ^71^ ^]^ Considering the wearable flexibility and charge transfer characteristics, flexible triboelectric acoustic devices commonly use polymer as charge exchange material, including fluorinated ethylene propylene (FEP),^[^ ^72^ ^]^ Kapton,^[^ ^73^ ^]^ polylactic acid (PLA), perfluoroalkoxy (PFA),^[^ ^74^ ^]^ polytetrafluoroethylene (PTFE),^[^ ^75^ ^]^ and so on. Metallic materials also contribute to the improvement in sensitivity when used as positive tribomaterials or electrodes.

### 2.5 Electromagnetic Mechanism

Electromagnetic acoustic transducers are commonly used in loudspeakers and microphones. External sound waves vibrate the coils and magnetic elements within the device at the same frequency and generate an induced current within the coils (Figure 2e). This process can also be reversed to produce the corresponding acoustic signal. Copper coils are extensively used in commercial acoustic sensors. Owing to their good ductility and conductivity, they can be used directly for machining extremely fine metal coils. Polydimethylsiloxane (PDMS)‐based flexible magnetic elements with uniformly distributed magnetic powders have potential for use in flexible acoustic sensors. Thus, electronic skins based on this mechanism can track subtle movements of the human skin, which makes it possible to monitor physiological representations of the human body during speech with a new level of precision. Considering the longevity of wearable devices, neodymium (NdFeB)^[^ ^76^, ^77^ ^]^ with its high remanent magnetization, high coercivity, and easy processing, has become the most commonly used material for magnetic powder particles and is widely used in various flexible magnetic sensing devices. The combination of TENG and magnetic materials further enhances the energy conversion efficiency, which can be used as an energy harvester for implantable devices.^[^ ^78^, ^79^ ^]^

### 2.6 Photoacoustic Mechanism

Light microscopy has higher spatial resolution than ultrasound imaging and provides richer information about tissues and organs in vivo. Photoacoustic microscopy has a deeper depth of penetration than optical methods, such as confocal microscopy, and better axial and lateral resolutions than ultrasound imaging. When a laser stimulates chromophores (e.g., hemoglobin, melanin, and lipids) in the body,^[^ ^80^ ^]^ these substances absorb the laser energy and produce a thermal effect that causes the thermoelastic expansion of nearby tissues, resulting in broadband ultrasound emission (Figure 2f). Because blood absorption is typically several orders of magnitude higher than that of the surrounding tissue, photoacoustic imaging provides sufficient endogenous contrast to visualize blood vessels. The resulting acoustic pressure propagation can be expressed as:^[^ ^81^, ^82^ ^]^

(5) $$\nabla^{2}p\left(\vec{r},t\right)-\frac{1}{v^{2}}\frac{\partial^{2}}{\partial t^{2}}p\left(\vec{r},t\right)=-\frac{\beta }{C_{p}}\frac{\partial }{\partial t}H\left(\vec{r},t\right)$$

where $H(\vec{r},t)$ is expressed as the thermal effect in a spatial location due to a light stimulus at a certain moment, *v* represents the speed of sound, *β* represents the coefficient of thermal expansion, and *C_p_* represents the constant‐pressure heat capacity. This equation requires the laser pulse width to be shorter than the thermal relaxation time as a precondition, which holds true most of the time. An opto‐acoustic imaging system consists of three main components: a laser resonator, an optical lens, and an acoustic detection system. Depending on the type of excitation, they can be categorized as photoacoustic computed tomography or photoacoustic microscopy. Photoacoustic computed tomography is not suitable for flexible wearable photoacoustic imagers because of the high power consumption of the laser resonator resulting from wide‐field scanning.^[^ ^83^ ^]^ Optical fiber are widely used in photoacoustic imaging to guide the excitation laser to the surface of the skin and to replace ultrasound receiver transducers. Wearable photoacoustics focuses on brain and subcutaneous vascular imaging, where three‐dimensional brain structures are constructed by identifying soft tissues in the brain with different optical absorption properties.^[^ ^84^ ^]^ In hemodynamic measurements, multiwavelength lasers can also be used for intravascular substance concentration measurements, thus enabling chemical monitoring under noninvasive conditions.^[^ ^85^ ^]^

### 2.7 Materials and Structure Design

Material selection and structural design of wearable acoustic devices directly determine their optimal use. The synergy between the two is significant. It is difficult for a single material to satisfy the requirements of high‐performance and wearable form factors. Researchers favor composites constructed with multiple materials because they can combine the mechanical and electrical properties of different materials by intertwining. The commonly used piezoelectric, resistive, dielectric, and triboelectric materials, as well as their composite structures and application areas, are summarized in **Table** **1**.

**Table 1:** Active materials in wearable acoustic devices.

Acoustic mechanism categories	Representative materials	Pros	Cons	Applications	Electrical parameters range	Refs.
Piezoelectric	PZT, ZnO, PMN‐PT, AlN, PVDF, P(VDF‐TrFE), BTO and 1–3 composites	Self‐powered operation, broad bandwidth, high sensitivity	Complex signal process, high impedance, single sensing direction	Acoustic signal detection, energy harvester, ultrasonic transducer, artificial throat, underwater detection, loudspeaker	Sensitivity: −140 to −30 dBV; Power density: 0.2 µW cm^−2^ to 70mW cm^−2^	[49, 53, 89, 103, 104, 105, 106, 107, 108, 109, 110, 111, 112, 113, 114, 115]
Resistive	Graphene, carbon nanotube, silver nanowires (AgNW), gold nanowire (AuNW)	High conformability, easy signal readout, high sensitivity	High power consumption, limited bandwidth, temperature‐sensitive	E‐skin, artificial throat, voice detection	Gauge factor: 30–2100 Pa^−1^	[63, 65, 67, 99, 109, 116, 117, 118, 119, 120, 121]
Dielectric	P(VDF‐TrFE), graphene PMMA‐laminated diaphragm, single crystalline silicon, MXene, PFA	Broad bandwidth, Flat frequency response	Sensitivity to surrounding environments, external power supply, complex signal readout	Artificial throat, wearable microphone	*ε*_r_: P(VDF‐TrFE): 10–12; Graphene: 3–5; Si: 11.7; MXene: 100–10000	[55, 90, 122, 123, 124]
Triboelectric	Kapton, polyurethane(PU), PFA, PET, PVDF, P(VDF‐TrFE), FEP, PTFE, polyvinyl chloride (PVC)	Self‐powered, high sensitivity, broad bandwidth, variety of materials, simple structure	Low signal output, complex signal process, vulnerable to surrounding noise	Energy harvester, acoustic monitoring, voice detection, underwater detection, artificial eardrum	Output power: 1–35 mW; Sensitivity: −100 to 17 dBV	[31, 72, 74, 124, 125, 126, 127, 128, 129, 130, 131]

Piezoelectric single crystals or ceramics embedded in polymers form 1–3 polymeric materials with good flexibility and mechanical‐acoustic conversion properties that are widely used in human wearable ultrasound applications. The “1” refers to the connectivity between the piezoelectric blocks in the polymer matrix in only one direction, while the “3” means that the matrix is interconnected in all directions.^[^ ^86^ ^]^ A variety of processes can be used to fabricate 1–3 piezoelectric polymers, including dice‐and‐fill (DAF),^[^ ^87^ ^]^ injection molding,^[^ ^88^ ^]^ and deep reactive ion etching.^[^ ^89^ ^]^ Different approaches have been adopted to fabricate transducers that are suitable for different situations. The temperature, piezoelectric volume fraction, filler crystallinity, and alignment during the process affect the performance of the final composite. An improper material arrangement or an excessively low processing temperature can lead to a decrease in the overall dielectric constant.^[^ ^90^ ^]^ In the selection of inorganic piezoelectric fillers, Pb‐free piezoelectric ceramics replace PZT or PMN‐PT as sensing elements, considering health factors.^[^ ^91^, ^92^, ^93^ ^]^ For example, Wang et al. developed biocompatible potassium‐sodium niobate (KNN)/epoxy lead‐free sandwich porous 1–3 composites.^[^ ^94^ ^]^ The composite has more than three times enhanced *g_33_* and two times enhanced quality factor compared to a single KNN material, and can be integrated into a flexible substrate and used as an implantable energy receiver in deep brain stimulation.

The material structure of Processing sensitive materials into fibers and forming nanowire networks can cover a large area of the human skin. Its good tensile properties make it widely used in sensors in the form of electronic skins and flexible fabrics. A nanowire network can reduce the number of grain boundaries, thereby alleviating the obstruction during electron transport and maintaining the conductive properties of the material.^[^ ^14^ ^]^ Various processing techniques have been used to prepare nanowire networks. It is essential to mention that the problem of material fracture at higher strains was avoided. The deposition of nanowire materials onto prestretched substrates can impart better tensile properties to the devices. Various materials have been used to build these composites, with silver nanowires (Ag‐NWs) being widely used in body‐worn sensors owing to their good stretchability, conductivity, and antimicrobial properties.^[^ ^95^, ^96^ ^]^

Membrane structures have a large area and light mass, giving them the advantages of fast response time, high sensitivity, and low energy consumption. Membrane structures are used in a wide range of applications. A series of sensing membranes with different thicknesses and areas can be used to capture rich acoustic information; they can also be actively vibrated to emit ultrasonic signals for detection. When exposed to an acoustic field, the sensing membrane is mechanically subjected to resonant deformation, and the sensitive materials used in the membrane produce corresponding changes in the electrical parameters. This process can also be reversed to drive the membrane to produce the corresponding acoustic signal. In addition, membrane‐type TENGs made of organic materials can enable the monitoring of acoustic signals with performances related to pre‐stressing and acoustic pores in the electrodes. However, the influence of environmental factors (e.g., temperature and humidity) on the long‐term performance stability of devices in wearable scenarios must be further considered.

### 2.8 Flexible Matching Layer and Substrate

The acoustic sensor substrate enables the wearable sensor to adapt to strains caused by human movement and acts as a matching layer to couple the acoustic impedance of the human skin. Good adhesion is also required to ensure a seamless fit to body tissues to minimize the transmission energy loss. Matching layer materials are usually fabricated as polymers, and composite matching materials are deposited on the device surface by chemical vapor deposition. Polymers have relatively good acoustic compatibility with soft tissues and can minimize the use of acoustic coupling media. This also enhances the overall durability of the sensor. Commonly used substrate materials are multidimensional polyester‐based materials such as polyimide (PI),^[^ ^97^, ^98^, ^99^ ^]^ PDMS,^[^ ^100^, ^101^, ^102^ ^]^ and polyethylene terephthalate (PET). Although these organic materials exhibit good tensile properties and chemical stability, they are relatively sensitive to temperature changes and pose certain acoustic energy loss problems. Hydrogel materials have become an important choice for wearable sensors because of their good biocompatibility, acoustic impedance matching, and bioadhesion. Owing to the existence of a crosslinked network, the hydrogel can dissolve and retain a large amount of water. The presence of proteins, carbohydrates, and other natural components makes hydrogel materials flexible and acoustic, similar to human tissues. Therefore, the hydrogel substrate achieved the smallest acoustic energy loss; however, most of the stability of the performance time was shorter.

## 3 Bioacoustic Signal Detection

Acoustic signals are the most fundamental media for animals to communicate and detect. Humans have a better communication efficiency than other creatures and can express more complicated information. Owing to the rapid development of artificial intelligence in recent years, many researchers have developed different types of voice‐recognition algorithms and databases to establish a complete solution that can be adapted to every situation.^[^ ^63^, ^132^, ^133^, ^134^, ^135^, ^136^ ^]^ Compared to collecting speech data for AI recognition through common microphones, wearable acoustic sensors are more noise‐resistant because they are directly attached to the human skin to acquire speech signals.^[^ ^137^, ^138^ ^]^ The miniaturization and low power consumption also allow for a high degree of integration of wearable devices, making it easy to wear them on various parts of the human body. In addition, by monitoring physiological acoustic signals, such as the user's voice and breathing sounds, potential health problems, such as heart and respiratory diseases, can be detected promptly.

### 3.1 Mechano‐Acoustic In Vivo

**Figure 3:**
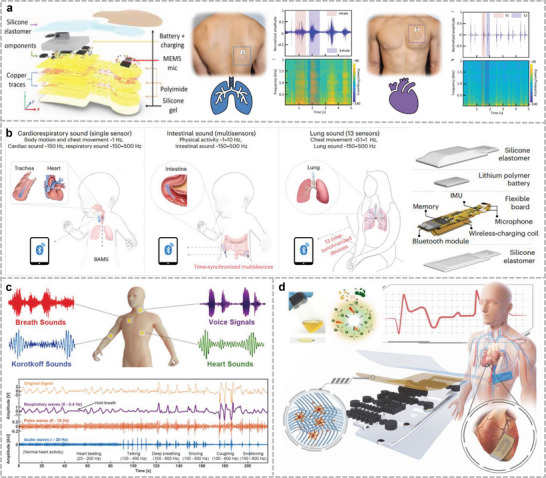
Wearable sensor for mechano‐acoustic signal monitoring. a) Fully portable MEMS stethoscope can be worn on the chest‐back. Reproduced with permission.^[^ ^28^ ^]^ Copyright 2022, American Association for the Advancement of Science. b) Wireless human mechano‐acoustic sensing network for monitoring heart, lung, and bowel sounds. Reproduced with permission.^[^ ^152^ ^]^ Copyright 2023, Springer Nature. c) Flexible sensing system based on folded double‐layer piezoelectric sensor for integrated detection of human acoustic signals. Reproduced under the terms of the CC‐BY license.^[^ ^156^ ^]^ Copyright 2023, Liuyang Han et al., Published by Wiley. d) Thin elastic conductive nanocomposites based on LIG micropatterning. Reproduced with permission.^[^ ^66^ ^]^ Copyright 2024, Springer Nature.

Some human organs, such as the heart and pulmonary respiration, work periodically. The acoustic signals potentially reflect the state of an organ. These acoustic signals were analyzed to determine their clinical value. For example, a cardiac murmur can reflect several potential types of heart disease, such as mitral valve stenosis and rheumatoid heart disease.^[^ ^139^ ^]^ Existing methods for monitoring body acoustic signals include stethoscopes,^[^ ^140^ ^]^ electrophysiological testing,^[^ ^141^, ^142^, ^143^ ^]^ and image and waveform recording.^[^ ^144^, ^145^, ^146^, ^147^ ^]^ The electrical signals of muscle groups at specific locations reflect the state of organ activity and can be captured through surface electrodes. Various forms of electrophysiological signals, like electrocardiography (ECG), surface electromyography (sEMG), and electrogastrography (EGG), are used in clinical diagnosis. These methods allow real‐time subject monitoring and can be used in adjustable positions as needed. Detection accuracy depends on the stability and specific location of the electrode attachment, which is only applicable to stationary individuals. MEMS devices with microphones can also be used to detect heartbeats. Lee et al. introduced a soft wearable stethoscope (SWS) for an automated disease diagnosis (**Figure** **3**a).^[^ ^28^ ^]^ Using a wavelet denoising algorithm, SWS successfully distinguished four types of lung diseases: crackle, wheeze, stridor, and rhonchi, with ≈95% accuracy. Because most bioacoustic signals, such as heartbeat, respiration, and guttural sounds, have a low frequency (<10 Hz) and intensity, the microphone cannot fully cover their bandwidth. To achieve complete monitoring of human bioacoustic signals, an additional sensor with a low resonant frequency is required to broaden the detection bandwidth.

With the development of microelectronics and micro‐/nanofabrication technologies, many motion sensors have been miniaturized and integrated into flexible circuits, leading to numerous devices for human acoustic signal monitoring.^[^ ^103^, ^148^, ^149^ ^]^ Accelerometers are one of the good choices for low‐frequency motion detection. Liu et al. proposed a mechanical acoustic–electrophysiological sensor device using miniaturized low‐power accelerometers.^[^ ^23^ ^]^ It enables the accurate recording of natural human body signals and also demonstrates the ability to be used as a voice interface for game operations. Gupta et al. proposed a capacitive triaxial accelerometer combined with a contact microphone to broaden the bandwidth, encapsulate the sensor's performance independently of environmental factors, and respond only to vibrations of the attached surface.^[^ ^150^ ^]^ Inertial measurement units (IMU) that measure three‐axis acceleration and angular velocity provide more comprehensive physiological movement information than accelerometers. By integrating an IMU into a flexible electronic platform, Lee et al. achieved a wide range of skin accelerations and whole‐body motion monitoring.^[^ ^151^ ^]^ Based on the results of the measurements of various daily motions, such as locomotion, respiration, and cardiac activity, they explored frequency‐domain analysis and machine learning to obtain real‐time recordings of essential vital signs. Yoo et al. demonstrated a wireless broadband mechano‐acoustic sensing system.^[^ ^152^ ^]^ This system uses an IMU and a pair of microphones (facing and back to the body, respectively) to enable the simultaneous acquisition of acoustic signals and differentiation using acoustic separation algorithms (Figure 3b). This device can assist in the clinical diagnosis of potential pulmonary respiratory diseases and in monitoring postoperative pulmonary function recovery.

Although the emergence of flexible circuits allows conventional sensors to be adopted in the design of conformal electronics, it is difficult to fully fit the accelerometer to the curved skin surface. Therefore, several materials with electromechanical properties have been fabricated.^[^ ^153^, ^154^ ^]^ Sensors with mechanoelectrical conversion effects, such as piezoelectric or piezoresistive sensors, can be used without an additional energy supply compared to conventional sensors. The difference between the high velocities originating in the arterial wall and the velocity of the pulse wave propagation results in highly nonlinear propagation, transferring wave energy from considerably low, barely audible frequencies to higher frequencies of audible content.^[^ ^155^ ^]^ Han et al. invented a folded double‐layer piezoelectric sensor and sensor array to record a wide range of vital signs (Figure 3c).^[^ ^156^ ^]^ The shielded and protected encapsulation layer offers the advantage of high‐fidelity physiological signals for various potential clinical applications. The utilization of materials with a certain flexibility can exceed the limitations of rigid sensors. Lu et al. developed a thin elastic conductive nanocomposite by transferring laser‐induced graphene (LIG) onto a hydrogel film (Figure 3d).^[^ ^66^ ^]^ With an ultrathin and adhesive polyvinyl alcohol (PVA)–phytic acid (PA)–honey (PPH) hydrogel as an interlayer of LIG, the intrinsic stretchability increased from 20% to 110%, which makes it possible to develop patches capable of MA signal monitoring in the presence of motion. Chen et al. fabricated an ultrafast‐response/recovery piezoresistive pressure sensor using DNA‐like double‐helix yarns.^[^ ^157^ ^]^ The double‐helix structure shows a significant improvement in the control of variation, linearity, and hysteresis without external torque or elastomers, which allows quicker responses to abnormal signals to follow the heartbeat more promptly. Ha et al. built hair‐thin, skin‐soft, and a highly stretchable PVDF vibration‐sensing e‐tattoo to detect high‐fidelity seismocardiography (SCG).^[^ ^112^ ^]^ By integrating Au electrodes and PVDF sensors on a substrate, the e‐tattoo can synchronously measure the ECG and SCG.

The fabrication of electromechanical and capacitive detectors requires complex processes, resulting in high manufacturing costs. Triboelectric acoustic sensors have attracted increasing attention from researchers owing to their strong conversion ability under low‐vibration conditions and low production costs. However, the sensitivity of triboelectric acoustic sensors changes under different pressure levels; stabilizing and increasing this parameter under low‐intensity conditions is the first challenge. Peng et al. built a breathable, biodegradable, and antibacterial e‐skin based on all‐nanofiber triboelectric nanogenerators.^[^ ^158^ ^]^ E‐skin has been prepared from silver nanowires (Ag‐NWs) sandwiched between poly(lactic‐hydroxyglycolic acid) (PLGA) and poly(vinyl alcohol) (PVA). Each layer comprises a random intercross of nanowires, and this combination creates thousands of micropores. This structure provided a specific surface area for contact charging and pressure responses.

Monitoring devices for human mechanical acoustic signals require the following considerations from a design standpoint: First, the device needs to fit into several key acquisition areas of the human body to obtain clear acoustic signals; therefore, the flexibility of the device should be able to conform to the curved surface of the skin at these locations. Second, because most human acoustic signals have low frequencies and amplitudes, the selected sensor must have sufficient sensitivity and response bands (**Table** **2**).

**Table 2:** Common mechano‐acoustic sensing approaches.

Sensing categories	Monitoring methods	organs	Pros	Cons
Stethoscope	Stethoscope^[^ ^140^, ^146^ ^]^	Heart, Lung, Intestine, Stomach	Convenient, fast, real‐time, multifunctional	Experience‐dependent, poor sound quality, limited information, difficult to quantify
Image and waveform recording	Echocardiography^[^ ^159^, ^160^, ^161^ ^]^	Heart	Real‐time, radiation‐free, multi‐modal, accurate measurements	Patient size, specialized operator, expensive
	Phonocardiogram (PCG)^[^ ^162^, ^163^, ^164^ ^]^	Heart	High sensitivity, quantitative analysis, complementary tool for stethoscope	Quiet environment, specialized equipment needs, limited availability of information and applications
	Pneumography^[^ ^165^, ^166^ ^]^	Lung	High sensitivity, quantitative analysis, complementary tool for stethoscope	Quiet environment, specialized equipment needs, limited availability of information and applications
	Acoustic Spectral Analysis^[^ ^167^, ^168^, ^169^, ^170^ ^]^	Heart, Lung, Intestine	High sensitivity and accuracy, automated and repeatable, long‐term monitoring	Equipment cost and complexity, high data processing requirements, quiet environment
Electrophysiological testing	Electrocardiogram (ECG)^[^ ^141^, ^171^, ^172^ ^]^	Heart	Real‐time monitoring, simple methodology, widely applicable	Limited structural information, short‐term recording
	Surface Electromyography(sEMG)^[^ ^173^, ^174^, ^175^ ^]^	Lung, Muscle	Specific muscle group attaching, real‐time monitoring, multi‐scenarios applicable	Indirect monitoring and evaluation, low accuracy, motion artifact
	Electrogastrography(EGG)^[^ ^176^, ^177^, ^178^, ^179^ ^]^	Stomach	Non‐invasive, continuous and dynamic monitoring	Low diagnosis accuracy, vulnerable recording process, lack of structural information

### 3.2 Gesture Recognition Through Acoustic Sensor

The fingers and arms are the most flexible joints in the body, and limb and hand movements are important for human expression. Wearable sensors collect information regarding different limb movements to create a database. AI algorithms analyze these movements to enhance the speed and accuracy of sensor recognition. Currently, most wearable sensor devices used for gesture recognition recognize gestures based on piezoresistive effects.^[^ ^180^ ^]^ These sensors were attached to the surface of the human hand to obtain the degree of deformation of each knuckle and construct gestures in virtual reality (VR). Owing to the high flexibility of the human hand, the performance of piezoresistive materials can be degraded by fatigue, which further reduces the recognition accuracy.^[^ ^148^ ^]^ Although TENGs have a remarkably high sensitivity for recognizing subtle changes in the hands, their output charges are more complex and unstable.^[^ ^129^, ^181^, ^182^ ^]^

**Figure 4:**
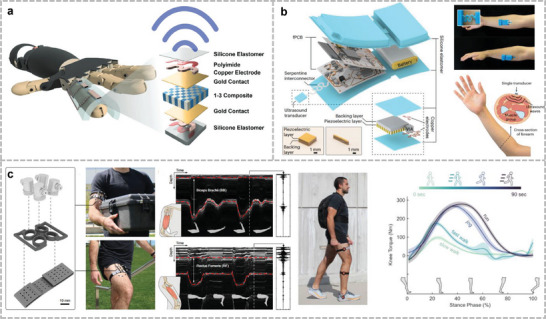
Gesture recognition through ultrasound method. a) Stretchable ultrasound array for contactless gesture recognition with small distance between each PZT composite. Reproduced under the terms of the CC‐BY license.^[^ ^40^ ^]^ Copyright 2024, Ankan Dutta et al., Published by Wiley. b) A fully integrated wearable ultrasound echomyography system that can be attached to the skin for long‐term accurate wireless monitoring of muscles. Reproduced with permission.^[^ ^186^ ^]^ Copyright 2024, Springer Nature. c) Monitoring change of muscle thickness during different movements through A‐mode ultrasound to estimate the torque of joint. Reproduced under terms of the CC‐BY license.^[^ ^41^ ^]^ Copyright 2024, Yichu Jin et al., Springer Nature.

Ultrasound signals can propagate through the body and air. There are two ways to recognize gestures with ultrasonic signals: One is to emit acoustic signals in air and determine the gesture based on the time of flight of the echo. This approach does not rely on the mechanical deformation of the sensitive element, and therefore, has a longer lifetime. Dutta et al. fabricated stretchable ultrasonic arrays with a close alignment using 1–3 PZT and PI surface charge engineering (**Figure** **4**a).^[^ ^40^ ^]^ This device has a distance of 20 µm between neighboring transducer units and can achieve high electromechanical coefficients of nearly 0.75 at 4 MHz. Based on this performance, multimodal acoustic signals can be generated to recognize complex hand movements such as punching, waving, sliding left and right, and opening and pinching at a distance of 7 cm. The second recognition method is based on changes in the small arm muscles under different hand movements. Various classification models have been adopted to classify muscle activity into different types of gestures.^[^ ^183^, ^184^, ^185^ ^]^ Although this method requires larger learning samples to improve the recognition accuracy compared to recognizing hand contours through ultrasound, the cost of the device is relatively lower, and there is no location restriction for hand recognition. report a fully integrated wearable echomyography system, which can be attached to the skin and provides accurate long‐term wireless monitoring of muscles (Figure 6b).^[^ ^186^ ^]^ They developed a deep learning algorithm for gesture recognition based on this system, correlating single‐sensor RF data from forearm muscles with hand gestures to accurately and continuously track 13 hand joints with an average error of only 7.9°. Similarly, Qu et al. used two PMUT chips as ultrasound probes and placed them orthogonal to each other to recognize small arm muscle changes under different gestures.^[^ ^187^ ^]^ The probes were encapsulated by an Ecoflex material to achieve lighter weight and power consumption compared with traditional transducers. A classification model was trained by collecting echo signals from the ultrasound and training the classification model based on the signals under 12 different gestures. This study achieved a recognition accuracy >5%. Jin et al. used ultrasound transducers to detect thigh and arm muscle groups and estimated the torque during various activity states based on type A ultrasound (Figure 4c).^[^ ^41^ ^]^ For the torque estimates generated in controlled situations, the average error was less than 7.6%, and the coefficient of determination was more significant than 0.92. Researchers used this strategy to test a range of complex physical activities in unconstrained situations, including cycling, weightlifting, and running. This study provides new insights into muscle function and exercise biomechanics, with potential injury prevention and rehabilitation applications.

### 3.3 Voice Recognition

Speech is the most important interaction method for human beings and is an important research direction in human–computer interaction. Traditional microphones for real‐time speech recognition are limited by sound quality, distance, orientation sensitivity, environmental noise interference, and other shortcomings that make it difficult to achieve accurate recognition. Although these limitations can be overcome using high‐performance microphones, noise reduction algorithms, and other methods,^[^ ^188^ ^]^ these methods are cost‐effective and have potential feature loss after post‐processing, resulting in a reduction in recognition accuracy. Microphones manufactured using MEMS technology have the advantages of small size, low power consumption, and noise elimination, permitting high‐quality recordings in small spaces.^[^ ^63^, ^127^, ^189^, ^190^ ^]^ Integrating the chip into a flexible system allows the microphone to capture high‐intensity acoustic signals with an optimal signal‐to‐noise ratio, thereby minimizing user actions for voice commands. In addition, the encapsulated microphone can maintain stable reception under a variety of complex conditions such as high humidity and strenuous activity. Lee et al. designed a capacitive acoustic diaphragm sensor with an improved accuracy for sound detection and a wider bandwidth.^[^ ^191^ ^]^ This sensor was composed primarily of silicon‐based materials. Owing to the low Young's modulus of the polymer and the optimized processes, the diaphragm contains less residual stress and shows higher sensitivity than metal‐based acoustic sensors. Lin et al. developed a waterproof acoustic sensor for wearable HMI.^[^ ^192^ ^]^ Acoustic vibrations cause a triboelectrification effect between PTFE and graphite, converting mechanical energy into electrical signals. This method has a high detection sensitivity of 3.2 mV Pa^−1^ in the 0.1–5 kHz and with the help of the AI algorithm, this sensor achieved a recognition accuracy of 98%. Fan et al. developed a bendable paper‐based triboelectric nanogenerator for acoustic energy harvesting.^[^ ^193^ ^]^ The ultrathin microhole structure significantly improved the response speed and sensitivity. Its wide frequency bandwidth allows it to record the sound of a speaker and collect acoustic energy that can charge a capacitor at a rate of 0.144 V s^−1^. The rollability of such a sensor can support a rigorously symmetrical structure and maintain consistent acoustic response performance in all directions.

**Figure 5:**
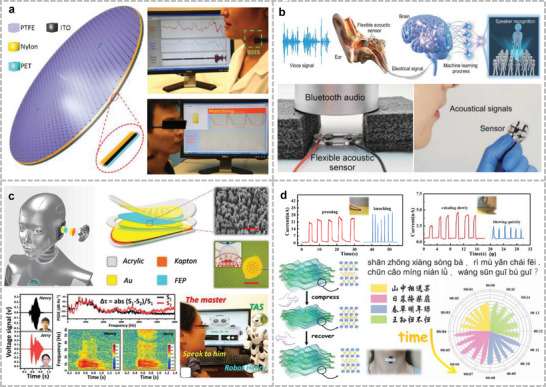
Wearable auditory sensors for speech recognition. a) Self‐powered eardrum‐inspired membrane sensor for arterial pulse wave monitoring and laryngeal microphones. Reproduced with permission.^[^ ^24^ ^]^ Copyright 2015, Wiley. b) New speech recognition platform developed with highly sensitive MXene/MoS_2_ flexible vibration sensor. Reproduced with permission.^[^ ^199^ ^]^ Copyright 2022, Springer. c) Highly sensitive self‐powered triboelectric acoustic sensors for robotic electronic hearing systems. Reproduced with permission.^[^ ^125^ ^]^ Copyright 2018, American Association for the Advancement of Science. d) A paper‐based pressure sensor prepared from MXene/bacterial cellulose film with a three‐dimensional barrier layer structure. Reproduced with permission.^[^ ^197^ ^]^ Copyright 2022, American Chemical Society.

In contrast to traditional microphones, flexible acoustic sensors can be attached directly to the skin. They effectively capture speech signals, particularly when placed in the throat, where the acoustic pressure is the highest. Lee et al. invented a flexible skin‐attachable sensor to record neck skin vibrations and found a linear relationship between it and SPL.^[^ ^194^ ^]^ Introducing polymers with low stiffness and damping properties achieves high sensitivity and a flat frequency response. In noisy and masked situations, this capacitive device excludes interference and exhibits a successful performance in several voice‐recognition applications. In addition to the audible sounds emitted by the vocal cords, many physiological activities are amplified and emitted by the larynx, some of which occur at frequencies below the lower line of the vocal band, and the detection of these signals is of considerable clinical diagnostic importance.^[^ ^195^, ^196^ ^]^ Yang et al. developed a self‐powered active sensor inspired by the structure of an eardrum with a lower bandwidth limit of 0.1 Hz (**Figure** **5**a).^[^ ^24^ ^]^ The sensor can continuously detect the throat sound signal in noisy environments when attached to the larynx and also continuously monitor the low‐frequency pulse wave of the human body, without the need for an external power supply. Su et al. built a neotype MXene/BC film pressure sensor using common paper as a flexible substrate (Figure 5d).^[^ ^197^ ^]^ This sensor can monitor several human activities, such as coughing, swallowing, and neck motion. Further, it can recognize different languages through neck vibrations during speech, as well as perceive air pressure to acquire environmental acoustic waves.

Owing to the development of artificial intelligence, the establishment of efficient and accurate artificial hearing systems has become an important research direction for bionic humans. Researchers have developed various bionic structures that are similar to the eardrum or cochlea of the human ear as auditory sensing elements for bionic humans. Such bionic sensors can be applied to robots as electronic ears to capture surrounding sound signals and correctly recognize human commands, thereby improving the intelligence of the robot.^[^ ^198^ ^]^ Recently, the emergence of bionic ears based on the triboelectric effect has provided a possible solution to these problems. Guo et al. built a self‐powered triboelectric auditory sensor as an external electronic hearing system for intelligent robotic manipulation (Figure 5c).^[^ ^125^ ^]^ This bioinspired eardrum has a broad bandwidth ranging from 100 to 5000 Hz and can customize the personal frequency response by adjusting the annular/sectorial inner boundary architecture. Sun et al. utilized the LIG fabrication method to integrate a TENG‐based microphone and a TA‐based loudspeaker, which broadened the frequency response (20 Hz to 20 kHz) and increased the sensitivity to 4500 mV Pa^−1^.^[^ ^39^ ^]^ The recognition accuracy of human speech content, identity, and emotion reached 99.66% and 96.63% for the training and testing sets, respectively, indicating better performance and broad development prospects. Chen et al. used a MXene/MoS_2_ flexible vibration sensor for speaker identification (Figure 5b).^[^ ^199^ ^]^ With a high sensitivity (25.8 mV dB^−1^) and broad frequency response (40–3000 Hz), a dataset was built and transformed into frequency‐domain signals and achieved a high recognition rate of 99.1%. Han et al. built a flexible piezoelectric acoustic sensor with a multi‐resonant frequency band.^[^ ^200^ ^]^ The seven channels used had different maximum relative responses, ensuring relatively high sensitivity to acoustic signals at all frequencies. In recognizing acoustic signals from the same loudspeaker, the device exhibited a 75% lower error rate than commercial MEMS microphones.

Speech data are the fuel for speech recognition, and their quantity and quality determine the accuracy and diversity of recognition, respectively. Accurate speech recognition relies not only on acoustic sensors with excellent performance to capture signals but also on the amount of data used for machine learning and the algorithms used to ensure accurate recognition. In addition to enriching the number of training samples by collecting a large amount of speech information, the multiscale acquisition of the same speech is an important way to improve recognition accuracy. Acoustic sensors fabricated using the MEMS technology exhibit excellent improvements in terms of sensitivity, bandwidth, and energy consumption. These enhancements allow researchers to use artificial intelligence algorithms to recognize specific information from speech, such as emotions and speaker identity.^[^ ^201^ ^]^

### 3.4 Artificial Throat

**Figure 6:**
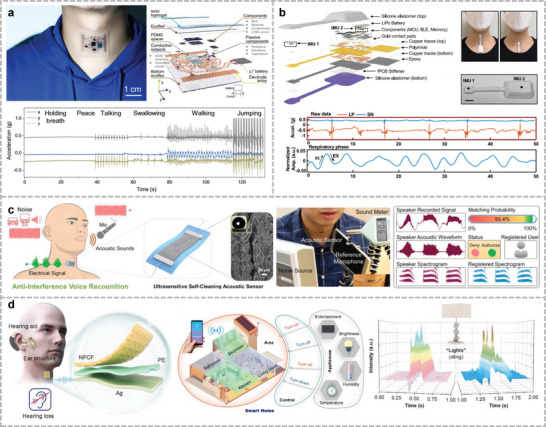
Artificial throat acoustic sensor. a) Standalone stretchable wearable platform that measures laryngeal vibration and muscle activity. Reproduced under the terms of CC‐BY license.^[^ ^208^ ^]^ Copyright 2023, Hongcheng Xu et al., published by Springer Nature. b) Flexible wireless device for monitoring physiological activity in the larynx such as breathing and swallowing. Reproduced under the terms of CC‐BY license.^[^ ^203^ ^]^ Copyright 2022, Youn J. Kang et al., published by Springer Nature. c) Attachable speech recognition system based on reduced graphene oxide/PDMS composite film with biologically inspired microcracks and layered surface textures. Reproduced with permission.^[^ ^42^ ^]^ Copyright 2019, American Chemical Society. d) Self‐powered artificial throat for the deaf population in a smart home scenario and recognizing process of common words. Reproduced with permission.^[^ ^204^ ^]^ Copyright 2019, Elsevier.

The larynx is the primary organ involved in pronunciation. Throat‐removal surgery causes patients with laryngeal diseases to lose their ability to speak. The implantable artificial larynx preserved the patient's laryngeal vocalization using a nickel‐titanium shape memory alloy as the epiglottic skeleton material. However, this method requires another surgery to install the device; this is not only expensive but also limited by the number of hospitals that can perform such surgery. An electronic device with flexible connections and a substrate that can be attached to the skin is a promising, nonsurgical, and cost‐effective solution for long‐lasting vocalization.^[^ ^63^, ^95^, ^137^ ^]^ This artificial throat, placed on the skin around the throat, mainly captures muscle activity associated with the articulation of various words and sounds. The attachable sensor translates the laryngeal muscle activity or skin deformation during speech into electrical signals. These signals are correlated with the corresponding linguistic information, and by combining them with machine‐learning classification algorithms, the method inputs the converted speech into digital devices with speech‐recognition functions. This will help users who have difficulty speaking because of vocal cord injuries or other reasons to communicate. In addition, in applications where swallowing and breathing are monitored in real time, this mechanical acoustic sensor acquires the phasic characteristics of swallowing and breathing by monitoring the movement of the skin surface of the neck and adjacent areas. Xu et al. designed a stretchable wireless sensing platform to measure throat activity (**Figure** **6**a).^[^ ^202^ ^]^ The platform uses modified composite hydrogels as surface electromyography (sEMG) electrodes to ensure synchronized deformation of the skin and accelerometers to acquire body motion signals. Measurements of 13 features/states during laryngeal activity (including several phonemes and activities, such as drinking and swallowing) were processed with a classification accuracy of 98.2%. Kang et al. developed a set of wireless wearable sensors to restore normal laryngeal function in patients with dysphagia (Figure 6b).^[^ ^203^ ^]^ The interference of motion artifacts was eliminated by differentially aligning the two IMUs to the neck and upper chest. Algorithms designed to analyze the swallowing and breathing activities were developed and used to monitor the subjects’ daily activities. Compared with standard clinical devices, the system achieves similar measurement fidelity without interfering with the patient's activity. Inspired by spider and lotus leaves, Le Dinh et al. presented a self‐cleaning, ultrasensitive, and ultrafast response skin‐attachable acoustic sensor based on a reduced graphene oxide/PDMS composite film (Figure 6c).^[^ ^42^ ^]^ The gap displacements inside the jagged microcracks were amplified by high‐frequency acoustic vibrations, resulting in an enhanced probability of disconnecting and reconnecting these cracks. This phenomenon allows the sensor to acquire a high measurement coefficient, low detection limit, and fast response. Zhao et al. designed a MEMS that senses mechanical displacements by utilizing electromagnetic induction.^[^ ^77^ ^]^ The device consisted of annular origami magnetic membranes and a multilayer flexible coil suspended at the center. This sensor can reach the bandwidth from 1 Hz to 10 kHz and offer sensitivity of 0.59 mV µm^−1^@ 1.7 kHz. When attached to the skin of the larynx, it converts human speech vibrations into electrical signals via electromagnetic induction. The corresponding electrical signals in different expressions can be recorded to realize a language recognition function.

Current smart devices increasingly support voice command control; nonetheless, vocally impaired individuals need to seek alternative control methods. Therefore, they cannot fully benefit from these technological advancements. The muscle activity of the throat is a viable approach as a substitute for voice commands through sensors in the form of an artificial larynx. Zhao et al. developed a self‐powered triboelectric vibration sensor (STVS) for hearing impairments to achieve speech recognition in this population (Figure 6d).^[^ ^204^ ^]^ The TENG consists of a woven structure of a nanocellulose film as a vibration‐sensitive component, maintaining excellent sensitivity over a wide frequency band range. The use of a Context Recognition Model (CRM) enables the real‐time recognition of 17 words commonly used in smart home scenarios, which can achieve 92.3% recognition accuracy. Che et al. presented a wearable, self‐powered sensing actuation system based on soft magnetic elasticity.^[^ ^76^ ^]^ Magnetic powder was attached to the polymer cells. When the cells are deformed, the magnetic particles change their magnetic flux owing to the torque, which generates an induced current in the metal coil. Through this phenomenon, the extralaryngeal muscle movement can be converted into a responsive electrical signal. Twenty‐five magnetic cells formed a flexible, stretchable array in the Kirigami structure to improve detection sensitivity. After classification by machine learning algorithms, prerecorded sound signals were played out through the drive section of the voice assist device to realize the corresponding speech‐signal output.

In addition to the acoustic emission function that drives vibrating membranes through piezoelectricity, capacitance, and other effects, the acoustic emission function through the thermoacoustic effect demonstrates even better acoustic emission performance. The thermoacoustic effect is achieved by the interaction between a solid medium and an oscillating fluid, which generates a time‐averaged heat flow in the direction of sound propagation within a certain range from the solid wall, and generates or absorbs acoustic energy in this region. Materials with a low specific heat capacity per unit area can quickly absorb and convert sound energy. In contrast, a low thermal inertia reduces the time required to reach thermal equilibrium, resulting in faster and more accurate sound emission. Graphene is the main material used in thermoacoustic sensing actuators, and loudspeakers based on this material have an ultra‐wide frequency response range and stable sound pressure output; however, the absence of additional actuator components remarkably simplifies the design of the device. Yang et al. developed a graphene‐flexible artificial throat with a honeycomb structure for mixed‐mode speech recognition.^[^ ^134^ ^]^ The sensor can detect that an artificial throat can generate 60 dB of sound pressure at a voltage of 5 V, with a frequency range of 100–20 kHz. The intelligent‐assisted speech technology established through this artificial throat restored the vocal ability of a volunteer patient with vocal cord disabilities, who was able to speak six sentences daily. An integrated auxiliary acoustic device was developed for transmission and reception. Compared with graphene materials, MXene‐based sound‐generating devices can produce higher sound pressure values at the same thickness. Gou et al. explored the applications of MXene materials in acoustics and developed Ti_3_C_2_ MXene nanoflakes.^[^ ^205^ ^]^ It shows excellent sound emitting ability, producing 68.2 dB SPL (f = 15 kHz). Kim et al. further improved the sound transmitting ability, which emitted an SPL of 74.5 dB (15 kHz) under a 0.5 W power supply.^[^ ^206^ ^]^ This loudspeaker was fabricated into a cylindrical and uniaxial kirigami structure for acoustic emission experiments, and the results show that the device can provide an omnidirectional stable output under tensile deformation conditions, demonstrating the potential of this material for artificial laryngeal applications. Metallic nanomeshes are capable of direct contact with the skin and various materials can be used as sensors. Qiao et al. established an EMG‐strain synergetic intelligent artificial throat.^[^ ^207^ ^]^ Au/PU was used as a strain sensor, combined with a Au nanomesh, and used as the electrodes of EMG to translate laryngeal muscle activities. Au/PVA, which was used as a sound emitter, achieved an SPL of 78 dB.

Artificial throats have promising applications in medical rehabilitation and provide intelligent solutions for patients with larynx damage. In the future, the demand for artificial throats will become increasingly personalized, with the devices customized according to individual needs and habits, and offering more natural and clearer voices. Therefore, improving the voice quality of an artificial throat to bring it closer to a natural voice effect is a leading research challenge. In addition, the wearability, durability, and manufacturing cost of artificial throats are the main factors restricting their popularity.

### 3.5 Silent Speech

Silent speech is a method of recognizing changes in the organs or skin during speech to determine what is intended to be said. Recognizing phonemes by analyzing facial movements and mouth shapes during speech offers considerable support to individuals with vocal impairments, such as damage to the vocal cord, larynx, and tongue. Silent speech recognition can be divided into two main categories: contactless and contactless. The contactless method is mainly based on the camera's visual signal to obtain facial changes, which requires users to maintain full vision of their face under the camera.^[^ ^209^, ^210^, ^211^, ^212^, ^213^ ^]^ Even though researchers try to capture the image through smartphones or specific devices continually, the light condition and camera angle still plague the utilization of such an approach, and the motion of the body will cause the blurred image. Using lidar with high frequency and high directionality is another effective contactless method; however, it is limited by power consumption, which cannot guarantee a long period of monitoring. The contact‐based method is achieved by directly mounting a sensor on the face, neck, tongue, or other speech motion organs or muscles.^[^ ^214^, ^215^, ^216^ ^]^ The main challenges in developing contact silent speech devices are how to achieve sufficient flexibility to avoid a sense of obstruction during use, sufficiently low power consumption to ensure prolonged operation, and sufficient sensitivity to capture the change signals in as much detail as possible.

**Figure 7:**
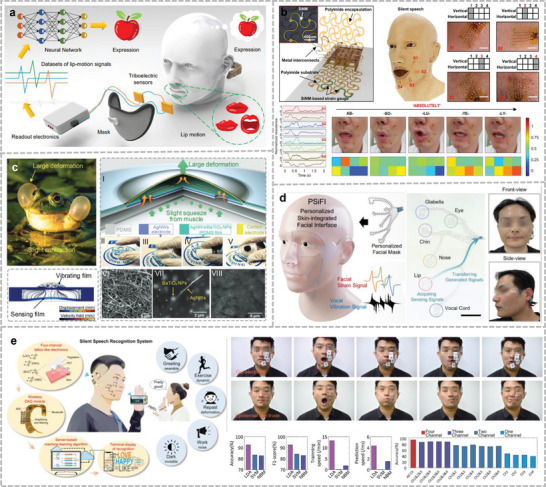
Silent speech recognition system. a) Self‐powered flexible triboelectric lip recognition system based on trained dilated recurrent neural network model for prototype learning. Reproduced under the terms of CC‐BY license.^[^ ^219^ ^]^ Copyright 2022, Yijia Lu et al., published by Springer Nature. b) Serpentine mesh tattooed strain transducer network fitting around the lips. Reproduced under the terms of CC‐BY license.^[^ ^221^ ^]^ Copyright 2022, Taemin Kim et al., published by Springer Nature. c) Real‐time micro‐movement of masticatory muscles into HMI inspired by the croaking behavior of frogs. Reproduced under the terms of CC‐BY license.^[^ ^230^ ^]^ Copyright 2021, Hong Zhou et al., published by Wiley. d) Personalized Skin‐Integrated Facial Interface (PSiFI) for simultaneous detection and integration of facial expression and speech. Reproduced under the terms of CC‐BY license.^[^ ^232^ ^]^ Copyright 2024, Jin Pyo Lee et al., published by Springer Nature. e) All‐weather silent speech recognition interface that transmits biological data from tattoo‐like electrodes to machine learning in real time. Reproduced under the terms of CC‐BY license.^[^ ^222^ ^]^ Copyright 2021, Youhua Wang et al,. published by Springer Nature.

Lip language is the earliest and most common form of silent voice interaction. The physiological mechanisms of lip‐language recognition in humans are controversial, and the accuracy of recognition shows little improvement after long‐term practice, which is inefficient. With the development of flexible electronic technology, many miniature and attachable electronic sensors have been applied to lip recognition and have shown significant research value.^[^ ^217^, ^218^ ^]^ Lu et al. proposed a novel lip‐language decoding system that adopts self‐powered triboelectric sensors to capture the motion of mouse muscles (**Figure** **7**a).^[^ ^219^ ^]^ A mask was used to fix the positions of the sensors and break the spread path of the disease. A dilated recurrent neural network model can recognize a dataset of 20 words collected by the device and achieve an accuracy of 94.5%. Applications for lip translation, such as identity recognition and voice emission, have been developed based on this recognition system, demonstrating the feasibility of lip recognition. Dong et al. developed an EMG‐based lip‐language system utilizing self‐adhesive, translucent, and suitable dry electrodes, which incorporated Ag‐NWs to serve as a conductive interface with the skin surface, whereas the self‐adhesive, compliant substrate was made of d‐sorbitol‐modified waterborne polyurethane (WPU).^[^ ^220^ ^]^ This sensor is unaffected by environmental conditions and can be used with or without vocalization. Kim et al. built a silent speech system by installing four single‐crystal silicon‐based strain sensors near the lips and incorporating a 3D convolutional deep‐learning approach (Figure 7b).^[^ ^221^ ^]^ The strain sensors were designed as ultrathin‐mesh serpentine structures that can adapt to the rapid deformation of the lips during speech without adding an elastic layer. By randomly selecting 100 words and repeating them 100 times for two subjects and using these strain data in the training of the deep learning model, a final recognition accuracy of 87.53% was achieved, which is a significant improvement over the accuracy obtained using sEMG sensors of the same size (42.6%).

More comprehensive information can be obtained by capturing changes in the facial skin. Since human emotions are mainly reflected in changes in facial expressions, capturing facial information can be extended to the judgment of user emotions. The sEMG technology is the most direct method for obtaining changes in epidermal muscles. Current inherent EMG electrode materials, including wet gels (Ag/AgCl electrodes) and bulk metal electrodes, cannot seamlessly contact the skin, which decreases the sensitivity and SNR.^[^ ^222^, ^223^, ^224^, ^225^ ^]^ Tattooed EMG electrodes are tacky, flexible, and imperceptible.^[^ ^226^ ^]^ A transparent, breathable, antimicrobial, and waterproof dressing was used to cover the detection electrodes to ensure biosafety and compatibility of the sensor for long‐term wear.^[^ ^227^, ^228^ ^]^ Although EMG can be used to visualize changes in the facial skin or muscles, its signal strength is limited and is accompanied by a certain amount of noise, which makes signal processing laborious. Triboelectric sensors are sensitive to small mechanical deformations and can capture and respond to these weak changes, thereby providing high sensitivity. Such a sensor forms electrical signals through charge transfer between different materials, which reduces the sensor's energy consumption and extends its service life; therefore, it is widely applied in silent speech.^[^ ^229^ ^]^ Zhou et al. developed a bionic TENG‐based ultrasensitive self‐powered electromechanical (BTUSE) sensor inspired by frog croaking (Figure 7c).^[^ ^230^ ^]^ It can translate the microdeformation of the masseter muscle into instructions. By mimicking the structure of the frog's oral cavity and vocal sac, a flexible PDMS material was used to create a sensor film and a deformable vibrating film, amplifying the tiny fluctuations of the chewing muscles and converting them into electrical signals. Xu et al. used a zwitterionic hydrogel to convert pressure action into current through changes in the ionic transport efficiency, with a 5‐fold increase in sensitivity compared with nonionic hydrogels.^[^ ^231^ ^]^ The bionic gel was integrated into a laryngeal silent speech recognition system that could convert laryngeal vibrations into the corresponding speech signals. With a recognition accuracy of up to 95%, the system enables silent and covert communication between users and computers. Lee et al. developed a personalized skin‐integrated facial interface for recognizing human emotions (Figure 7d).^[^ ^232^ ^]^ Five strain TENG sensors were placed on the glabella, eye, nose, lip, and chin, and a vibration TENG sensor was placed on the neck to record the acoustic vibration. A CNN‐based classification model was used to analyze the collected multimodal data of facial expressions and speech signals and to obtain emotional information from the user. Communication with an attached emotional context in the VR environment was successfully implemented. Wang et al. developed an all‐weather speech strategy for silent interactions (Figure 7e).^[^ ^222^ ^]^ Facial skin deformations were obtained using electronic tattoo sensors attached to the face and converted into verbal commands using machine learning algorithms with a data acquisition module. The system adapted to large facial deformations and successfully recognized 110 words with a recognition accuracy of 92.64%.

Although these contact sensors can obtain more intuitive and accurate facial information, the collection of data to support AI algorithms requires the use of multiple sensors to obtain multi‐dimensional data and prolonged and extensive areas of skin attached to more or less uncomfortable areas. Dong et al. established soft magnetic skin to capture articulatory movements and enable the recognition of phonemes, word pairs, and sentences.^[^ ^233^ ^]^ Magnetic skin comprises silicone polymers and magnetic particles separated inside the polymers, which can seamlessly attach to the skin and have three different magnetization directions along the *x*‐, *y*‐, and *z*‐axes to monitor displacements and strains in real time. A silent speech recognition interface developed based on this device can support the control of smartphones and drones, demonstrating its potential for human–computer interaction.

### 3.6 Bioinspired Acoustic Sensor

**Figure 8:**
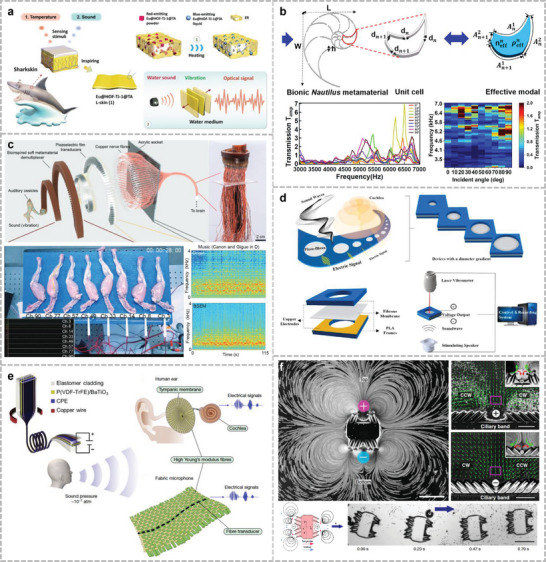
Bio‐inspired acoustic sensor. a) Underwater bionic ultra‐thin flexible sensors inspired by shark skin. Reproduced with permission.^[^ ^236^ ^]^ Copyright 2024, Wiley. b) Metamaterials with bionic nautilus structure for multi‐information fusion compressive sensing acoustic imaging device. Reproduced with permission.^[^ ^242^ ^]^ Copyright 2023, Elsevier. c) Bionic hearing system consisting of soft elastic metamaterials and piezoelectric transducer diaphragms. Reproduced under the terms of CC‐BY license.^[^ ^245^ ^]^ Copyright 2023, Hanchuan Tang et al., published by Wiley. d) Fiber membrane composed of piezoelectric nanofibers to sense acoustic vibration signals in different frequency bands by adjusting the diameter of the membrane. Reproduced under the terms of CC‐BY license.^[^ ^246^ ^]^ Copyright 2020, Giuseppe Viola et al., published by American Chemical Society. e) Sensitive elements using BTO/P (VDF‐TrFE) composites as piezoelectric fibers that can be woven into high elastic modulus fabric media for bionic eardrums and wearable acoustic transmitters and receivers. Reproduced with permission.^[^ ^252^ ^]^ Copyright 2022, Springer Nature. f) A micro‐robot with ciliated bands that mimics the surface of a starfish larva, with acoustic waves driving the cilia to vibrate. Reproduced under the terms of CC‐BY license.^[^ ^253^ ^]^ Copyright 2021, Cornel Dillinger et al., published by Springer Nature.

Mimicry of the physiological structure of natural organisms or their movements has always been a source of inspiration for scientific research. A wide range of biological structures and functions that have evolved in nature have the advantages of efficiency and flexibility, which are the best solutions in most scenarios. Many biologically inspired biomimetic results have been reported in the field of acoustics.^[^ ^234^, ^235^ ^]^ The acoustic properties and auditory mechanisms of marine organisms have aided the design and development of new technologies for various applications.^[^ ^236^, ^237^, ^238^, ^239^ ^]^ Artificial skin sensors have become a research hotspot in recent years, mimicking the structure of biological skin to sense external stimulus information such as temperature, haptic,^[^ ^240^, ^241^ ^]^ and chemistry of the surrounding environment.^[^ ^60^ ^]^ However, an electronic skin capable of sensing acoustic vibrations has not been reported in large‐scale studies. Inspired by sharks sensing acoustic vibrations in water through their skin, Xu and Yan developed Eu@HOF‐TJ‐1@TA L‐skin, an artificial skin sensor capable of sound‐temperature composite sensing (**Figure** **8**a).^[^ ^236^ ^]^ The sensor exhibited an ultra‐high maximal relative sensitivity (97.669% K^−1^) and ultra‐low minimal temperature uncertainty for sensing temperatures from 298.15 K to 358.15 K. The sensor can also be used to sense the temperature of the surrounding environment by mimicking the structure of biological skin. For temperature sensing from 298.15 K to 358.15 K, the sensor exhibits an ultra‐high maximum relative sensitivity (97.669% K^−1^) and ultra‐low minimum temperature uncertainty of 0.000 952 K, with maximum response frequencies of 400 Hz in air and 300 Hz in water.

The nautilus is favored by researchers because of its unique helical structure and good acoustic performance. The spiral structure can overcome the limitations of sensors in terms of bandwidth and directivity. Based on this advantage, Wang et al. proposed a bionic metamaterial multi‐information‐fusion compressive sensing acoustic imaging device for sound localization and recognition (Figure 8b).^[^ ^242^ ^]^ This bionic metamaterial replaces the traditional randomly coded structure design method with the regular spiral‐gradient geometric features of a nautilus. Introducing a multi‐information fusion approach enhances the compressed sensing acoustic imaging capability of CSAI to recognize and image multiple broadband sound sources in noisy interference environments, thereby demonstrating the potential of this device for acoustic sensing. Fiorillo et al. designed spiral piezoelectric polymer thin‐film sensors to generate and receive ultrasound.^[^ ^114^ ^]^ A PVDF spiral diaphragm blocks only the ends of the diaphragm, leaving it free to vibrate in air. Compared with common commercial piezoelectric polymer transducers, they exhibit omnidirectional ultrasonic emission and reception over a wide frequency range (20–80 kHz).

Spiral structures have also been observed in the human cochlea. Thousands of sensory cells (hair cells) are distributed across the basement membrane. When the basilar membrane vibrates, hair cells within the fluid generate electrical signals that are decoded and interpreted by the brain to transform them into perceivable sounds. Different locations of the cochlea pick up sounds at different frequencies, with the spiral, wide outer opening picking up high‐frequency sounds, and the inner end of the spiral picking up low‐frequency sounds. Based on this signal‐transformation mechanism, many researchers have used piezoelectric or triboelectric materials as sensing cores to develop bionic cochleae.^[^ ^243^, ^244^ ^]^ Tang et al. developed a bioinspired elastic metamaterial to reproduce the shape and function of the human cochlea (Figure 8c).^[^ ^245^ ^]^ Helically distributed hierarchical microstructures have a high refractive index, enable position‐dependent frequency demultiplexing for tenfold passive sound enhancement, and are capable of high‐speed parallel processing, supporting up to 168 acoustic/piezoelectric signal processing channels. The cochlear implant has a wide audible range of 150–12000 Hz and a passive analog output voltage of up to 2 V, enabling activation of the auditory pathway in mice without power. Viola et al. designed and fabricated acoustic loop transducers based on P(VDFTrFE) nanofibers using electrospinning technology, which were capable of generating electrical signals of up to 17 mV at low frequencies ranging from 100 Hz to 400 Hz (Figure 8d).^[^ ^246^ ^]^ A fibrous membrane consisting of biocompatible polymer piezoelectric nanofibers mimics the function of a basement membrane that selectively vibrates in response to different sound frequencies. Compared with aligned nanofiber sensors, random nanofiber sensors have a wider range of frequency selection and higher sensitivity, as evidenced by the downward shift in the resonance frequency and higher voltage output as the diameter of the circle increases. A new strategy for efficient, flexible piezoelectric acoustic sensor (FPAS) micro‐cone patterned arrays based on multicomponent lead‐free encapsulated crystalline rod niobate piezoelectric materials with morphological phase boundaries (MPBs) is reported by Xiang et al.^[^ ^247^ ^]^ The patterned micro‐cone arrays increase the contact area with acoustic waves and thus enhance the absorption of acoustic energy, with sensitivities as high as 150.63 mV Pa^−1^ and 39.22 mV Pa^−1^ cm^−2^. Bionic development of the human auditory system includes simulation of the tympanic membrane.^[^ ^248^ ^]^ Damage to the tympanic membrane due to various factors, such as infection, trauma, and noise exposure, is common. Developing a bionic acoustic device that can replace the tympanic membrane is crucial for individuals suffering from such issues.^[^ ^249^, ^250^, ^251^ ^]^ Wei et al. introduced a fabric‐like acoustic sensor consisting of a high Young's modulus textile yarn and thermally stretched composite piezoelectric fibers (Figure 8e).^[^ ^252^ ^]^ It can convert a weak pressure wave of 10^−7^ atm at the audible frequency into a low‐order mechanical vibration mode and then into an electrical signal. Fabrics with integrated fibrous acoustic transducers that also act as sound transmitters and receivers can establish bidirectional communication between the two fabrics, and clothing featuring these fabrics can be used as a stethoscope to detect real‐time heart sound signals.

Ciliated structures are found in human cells for acoustic perception and are also prevalent in prokaryotic and eukaryotic cells. Cilia are mainly divided into protocilia, which are the sensory structures of the cell, and motor cilia, which are used to drive the movement of the cell to navigate through fluids. Inspired by natural systems, researchers developed synthetic cilia and applied them to microfluidics and microrobotics for propulsion, fluid delivery, and mixing. Dillinger et al. mimicked the natural arrangement of ciliated ribbons on the surface of starfish larvae by ultrasonically activating synthetic cilia ribbons, which underwent small‐amplitude oscillations and generated a controlled fluid flow upon activation (Figure 8f).^[^ ^253^ ^]^ When ultrasound‐activated planar ciliated bands are tilted (+) toward each other on the support surface, the liquid is pushed away from the surface, simulating a source of flow. Conversely, when the cilia bands are angled away (−) from each other, the liquid is pushed towards the surface, simulating a flow channel. By arranging the “+” and “−” cilia bands next to each other, the micro‐robot is able to achieve unidirectional motion in the ultrasound field, and the incorporation of gas‐filled micro‐bubbles between individual cilia allows the robot to perform steering functions when driven by ultrasound alone. The robots demonstrate the potential for motion control within human blood vessels. Future control experiments will be conducted in animal architectures, and their performance in non‐Newtonian fluids such as blood, viscoelastic media, and shear‐thinning gels will be investigated.

## 4 Ultrasound Energy Supply and Communication

Human implantable devices are important components of medical devices; however, their miniaturization, biocompatibility, and power supply are still the main difficulties hindering their development. Ultrasound signals are widely used in organ and tissue imaging because of their excellent penetration and directivity. Recent advances in microenergy‐harvesting technology have expanded the use of ultrasound in implantable devices for energy supply and communication, eliminating the need for subsequent surgeries to replace batteries. In addition, the potential medical value of ultrasound in wound healing and nerve stimulation has recently been exploited. The acceleration of healing is achieved through various forms of ultrasound action (e.g., focused ultrasound,^[^ ^254^, ^255^, ^256^, ^257^ ^]^ transcranial ultrasound,^[^ ^258^, ^259^, ^260^, ^261^ ^]^ acoustic cavitation,^[^ ^262^ ^]^ etc.). This section focuses on specific use cases of wearable ultrasound devices developed for these applications and summarizes the highlights of their development and possible future challenges.

### 4.1 Energy Supply for Implanted Device

Implantable medical devices have become popular with the development of clinical therapeutic techniques whereby devices are fitted to specific parts of the body through surgical or other clinical interventions to perform specific functions. Implantable devices can be categorized into articulated tissue‐fitting structures and active devices.^[^ ^263^ ^]^ The active implantable devices are primarily used to monitor the physiological information of organs and assist in their normal functioning. For example, an implantable cardiac defibrillator can continuously monitor a patient's arrhythmia using electrodes. When an abnormal rhythm is recognized, the defibrillator automatically terminates the abnormal state through an electric shock, thus ensuring that the pacing function of the heart is normal.

**Figure 9:**
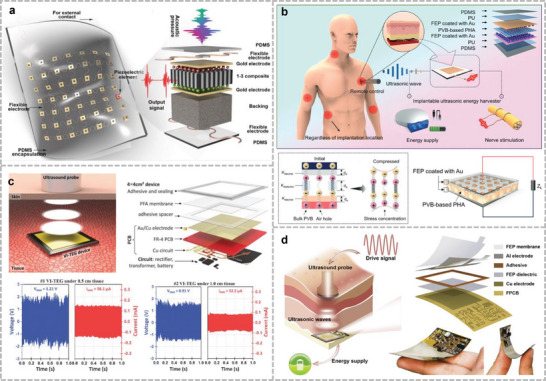
Ultrasound energy‐harvester for implantable device in vivo. a) Ultrasonic energy harvester arrays integrating a large number of piezoelectric active devices with multilayer flexible electrodes that produce continuous voltage and current outputs on planar and curved surfaces. Reproduced with permission.^[^ ^265^ ^]^ Copyright 2019, Elsevier. b) Multilayer piezoelectric with strain enhancement for in vivo energy harvesting, with parallel stomatal arrays introduced to improve energy efficiency. Reproduced with permission.^[^ ^266^ ^]^ Copyright 2022, Wiley. c) Implantable vibratory TENG with ultrasound‐induced micrometer‐scale displacement of polymer films to generate electrical energy through contacts. Reproduced with permission.^[^ ^74^ ^]^ Copyright 2019, American Association for the Advancement of Science. d) Integration into a flexible printed circuit board triboelectric nanogenerator using electrodes with optimized structural parameters improved output power by 66% and can provide a stable 1.8 V DC voltages. Reproduced with permission.^[^ ^31^ ^]^ Copyright 2022, Elsevier.

Active implantable devices require additional batteries to provide energy, and therefore require periodic surgeries to recharge or replace the batteries, which notably increases the risk of patient exposure to germs and other health hazards. The first involves harvesting the mechanical energy generated by the movement of organs in the body and converting it into electrical energy that can be used to support the continued operation of the system. Kim et al. developed an in vivo energy‐harvesting and wireless communication function using a high‐performance single‐crystal (PMN‐PZT) piezoelectric material.^[^ ^264^ ^]^ During this period, the kinetic energy of the pig's heartbeat was collected. An open‐circuit voltage of 17.8 V and a short‐circuit current of 1.74 µA can be generated. This method can perform self‐powered wireless communication data transmission. However, this method has limited energy stability, and energy can be generated from one body part to another. An external wireless transmission device can generate a stable energy supply field for internal devices that is more stable than the former and is not limited by body parts. The external energy supply is primarily provided through electromagnetic induction, light energy, and ultrasound. Compared to the other two methods, ultrasound has better penetration and directivity when passing through the body, and its nonionizing radiation nature makes it have less impact on human tissues; therefore, it is widely used in the energy supply of implantable devices. To expand the application of acoustic energy harvesting in implantable devices, the energy harvester should be form‐fitted to ensure a tight fit to the curved organ, especially in the case of the heart, which undergoes significant activity for a long period of time, to ensure that no detachment problem occurs. Jiang et al. developed a flexible piezoelectric ultrasonic energy collector array to generate continuous voltage and current (2 Vpp, 4 µA) under planar and curved ultrasound‐driven conditions (**Figure** **9**a).^[^ ^265^ ^]^ Different thicknesses of pork were placed between the ultrasonic transmission and collection patches under curved conditions, with up to 15% voltage attenuation in the thickest case. Wan et al. developed a strain‐enhanced multilayer piezoelectric device by introducing arrays of parallel‐connected air holes in the dielectric layer between the electrodes (Figure 9b).^[^ ^266^ ^]^ At an ultrasound probe setting of 25 mW cm^−2^, the device provides a significant peak output power of ≈13.13 mW and a short‐circuit current of ≈2.2 mA when implanted into a tissue of 5–10 mm, which is capable of meeting the power threshold required for implantable electronic devices.

Triboelectric generators based on membrane structures also exhibit significant potential for acoustic energy harvesting. Compared with the energy receiver based on the piezoelectric effect, the acoustic energy receiver based on the triboelectric principle generates electrical energy through periodic contact separation of the upper and lower membranes, which is relatively simpler in structure and therefore cheaper in terms of manufacturing cost. The basic mechanism of the large‐size membrane structure to collect acoustic energy is that the upper membrane is repeatedly contacted and detached from the bottom flexible electrode under the action of ultrasonic waves; charge transfer occurs in the process, and the separated charge flows into the circuit to drive the implanted parts to provide electrical energy. Hinchet et al. developed a transcutaneous capacitive triboelectric energy harvester that can effectively collect mechanical energy (Figure 9c).^[^ ^74^ ^]^ The ultrasound wave induces a 3.6 cm × 3.6 cm upper membrane that is made of PFA to produce micrometer‐scale displacement to contact Au/Cu electrode. The receiver can achieve a maximum voltage of 1.21 V and 0.91 V on a 40 megohm resistor in the case of 0.5 cm and 1 cm thick pork spaced apart for energy supply experiments with ultrasound at a frequency of 20 kHz and a power density of 1 W cm^−2^, respectively. Yang et al. first developed a flexible implantable device supplied with external ultrasound for the treatment of brain cancer.^[^ ^267^ ^]^ The device is excited by external ultrasound power to generate an alternating cancer therapy electromagnetic field that exerts biophysical forces on the molecules necessary for cell division, thereby disrupting tumor cell division and inducing apoptosis in dividing cells. Most large membrane structures use a single‐electrode membrane structure. However, this structure cannot effectively utilize the transmitted charge in one operating cycle. To improve the output power, Liu et al. used a two‐electrode TENG structure, enabling a higher output power production with lower impedance in the same area (Figure 9d).^[^ ^31^ ^]^ The device could supply an implantable medical device with a stable DC voltage of 1.8 V. This demonstrates the effectiveness of the device by applying significant electrical stimulation directly to the sciatic nerve of a bullfrog. Higher‐frequency ultrasound signals may have a higher energy density; therefore, having a correspondingly matched acoustic energy receiver for harvesting can enhance the energy transfer efficiency. Furthermore, smaller structures allow implantable devices to be more compact, thus reducing the interference of the device with the human tissue.^[^ ^25^ ^]^ Based on this feature, Sun et al. designed interlocking nanostructures to enhance the response to subtle strains capable of reaching a bandwidth of 10 MHz.^[^ ^268^ ^]^ The device collects low‐frequency ultrasound signals and powers implantable electronic devices. In the high‐frequency band, when integrated with a flexible electromagnetic coil, the device converts the encoding of high‐frequency acoustic signals into electrical energy, synchronizes them to the coil, and generates the corresponding encoded electrical signals, thus enabling in vivo and in vivo wireless transmission capabilities.

Despite remarkable progress in implantable acoustic energy harvesters, many challenges remain. First, improving the energy harvesting efficiency is a key issue. Although existing technologies have been able to achieve effective energy harvesting to a certain extent, a higher efficiency is still required to meet the energy demand of devices in practical applications. Rational integration and miniaturization of devices capable of harvesting mechanical energy from organ activity in the body should be considered to increase the output power as much as possible. Second, the distance between the device and the epidermis can vary owing to organ activity, which causes significant fluctuations in transmission efficiency; therefore, the bandwidth of the receiving device needs to be expanded to maintain maximum power. Finally, for implantable devices that must be surgically removed, materials with degradation properties should be developed to ensure that the body automatically degrades them by the end of the treatment cycle (**Table** **3**).

**Table 3:** Comparison of several typical ultrasonic energy harvester.

Structure	Ultrasound type	Frequency	Ultrasonic intensity	Output Power	Refs.
PFA membrane	Sine wave	20 KHz	3 W cm^−2^	6.8 µW	[74]
Hybrid‐piezoelectret	Pulsed sine wave	100 kHz	25 mW cm^−2^	154.6 µW	[266]
FEP membrane	Sine wave	28 KHz	–	35 µW	[31]
MEMS membrane	Sine wave	1 MHz	63 kPa	297 nW	[25]
Piezoelectric array	Sine wave	2.08 MHz	–	0.15 W cm^−2^	[269]
M‐gel generator	Pulsed sine wave	20 KHz	50 mW cm^−2^	21.8 µW	[270]
Piezoelectric ultrasonic energy harvester	Sine wave	370 kHz	1 mW cm^−2^	84.3 nW	[271]
wood‐templated piezoceramic	Sine wave	40 kHz	0.6 W cm^−2^	304 µW cm^−2^	[272]
1‐3 composite	Pulsed sine wave	304 kHz	–	63 µW	[273]

### 4.2 Ultrasonic Communication

A wide variety of physiological indicators are used to assess human health, and comprehensive real‐time access to these indicators can help accurately assess the health status of patients in a timely manner. Implantable medical devices can gather physiological information in the body that is difficult to obtain externally, making them a crucial component in building the Internet of Medical Things. However, there are difficulties in data transmission using implantable devices. Commonly used methods of communicating with implantable devices include wireless technologies, such as radio frequency identification (RFID),^[^ ^264^ ^]^ Bluetooth,^[^ ^274^ ^]^ and near‐field communication. These methods have some inherent disadvantages that cannot be well applied in human communication scenarios. For example, RFID is susceptible to interference and blocking, leading to limited communication reliability, and the Bluetooth method is relatively high in energy consumption, which tends to quickly consume the power of implantable devices. Ultrasound signals are used in implantable device communication applications owing to their low attenuation rate and high safety thresholds in the human body. Jiang et al. used flexible lead‐free piezoelectric arrays for ultrasound energy harvesting and communication.^[^ ^269^ ^]^ In the signal transmission test, 500 ms was set as the bit signal length, and the trigger signals were programmed to be aligned with the trigger signals of the time sequence in a high‐signal‐to‐noise‐ratio pulse sequence, which proved that there was no bit error in the method. It can be used in conjunction with transmission chips in future implantable bioelectronic systems for physiological monitoring and smart control applications.

**Figure 10:**
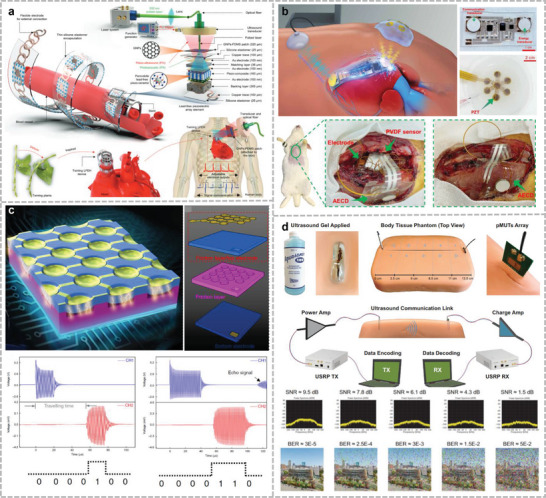
Ultrasonic transducer for in vivo communication. a) A novel design and implementation of a hybrid induced energy transfer strategy using photoacoustic (PA) and piezo‐ultrasound (PU) technology in a 3D twining wireless implant. Reproduced with permission.^[^ ^273^ ^]^ Copyright 2021, Royal Society of Chemistry. b) An implantable acoustic energy transmission and communication device (AECD) that maintains good ultrasound function after bending and provides intelligent monitoring and emergency treatment of the heart. Reproduced under the terms of CC‐BY license.^[^ ^275^ ^]^ Copyright 2021, Peng Jin et al., published by American Association for the Advancement of Science. c) Micro‐structured triboelectric ultrasonic device (µTUD), using a vacuum chamber to eliminate ambient effects. Reproduced under the terms of CC‐BY license.^[^ ^25^ ^]^ Copyright 2020, Chen Chen et al., published by Springer Nature. d) Lithium niobate piezoelectric micromachined ultrasonic transducers for high data‐rate intrabody communication. Reproduced under the terms of CC‐BY license.^[^ ^276^ ^]^ Copyright 2022, Flavius Pop et al., published by Springer Nature.

Unlike wireless communication outside the body, because of the directional nature of ultrasound, in vivo information transmission must consider the orientation of the receiver to ensure that the signal transmission strength is maximized. This means that when designing an implantable device, the location and orientation of its receiver, as well as the manner in which the transmitter emits, must be considered. One approach involves the use of multiple receivers to increase the coverage of the received signal. To address this issue, Jiang et al. drew inspiration from entangled plants and developed a 1 × 13 strip lead‐free receiver based on previous flexible receivers that can be wound around blood vessels; this structure allows the array to maintain a consistent output voltage in different orientations (**Figure** **10**a).^[^ ^273^ ^]^ Ring‐mounted focused piezoelectric transducers integrated with pulsed optical fibers with piezoelectric‐optoacoustic actuators generate hybrid acoustic sources for energy transfer and communication and demonstrate that optoacoustic‐based pulses with shorter signal lengths (2 µs) are more promising for communication applications, and also enable simple 64‐pixel black‐and‐white image transmission capabilities through a combination of time slots and channels. Peng et al. developed a thin, flexible, implantable system for simultaneous wireless recharging and communication simultaneously through ultrasound (Figure 10b).^[^ ^275^ ^]^ They explored a convenient ultrasonic focal method to increase the transmission ability and control the focusing position. The hole electronic circuits are encapsulated inside the PDMS material, which prolongs the use life and can be integrated with different types of sensors or actuators to transmit biosignals and execute emergency treatment if any abnormal symptoms occur. Chen et al. developed a TENG and used it for energy harvesting via a MEMS process, which increased the operating frequency to 1.17 MHz for the first time (Figure 10c).^[^ ^25^ ^]^ in addition, it could generate output voltages of up to 16.8 mV and 12.7 mV in oil‐immersed and pork‐tissue‐isolated cases, respectively. This study exploits the application of miniaturized devices for energy harvesting. The top membrane structure bent in contact with the bottom at only a few locations of maximum displacement. Pop et al. used PMUT arrays with different center frequencies combined to form a larger bandwidth (Figure 10d).^[^ ^276^ ^]^ Lithium Niobate, which has a higher piezoelectricity coefficient, was used to cover a deeper depth range and achieve a higher transmission rate, and a bitstream coding and decoding method was used to achieve an acoustic transmission function for color images.

### 4.3 Neural Stimulation and Recording

The human nervous system is one of the main systems that controls and regulates the body. A close connection exists with the endocrine system, which is regulated by the release of various hormones that affect physiological processes in the body. By stimulating neuronal activity, it is possible to modulate the function of the nervous system, reduce symptoms, improve the quality of life of the patient, and combine it with brain‐computer interfaces to enable the control of external devices.

**Figure 11:**
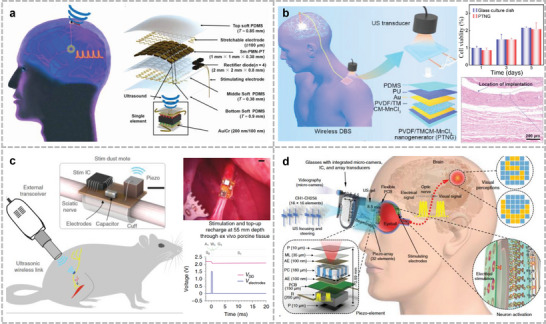
Implantable neural therapy and recording device stimulating through the ultrasonic method. a) Flexible piezoelectric energy harvester for deep brain electrical stimulation based on SM‐PMN‐PT single crystals converts collected acoustic energy into electrical stimulation. Reproduced with permission.^[^ ^35^ ^]^ Copyright 2022, American Association for the Advancement of Science. b) Ultrasound‐driven battery‐free neurostimulator based on a high‐performance PVDF/TMCM‐MnCl_3_ hybrid piezo‐triboelectric nanogenerator. Reproduced with permission.^[^ ^277^ ^]^ Copyright 2023, American Chemical Society. c) A wireless, leadless, battery‐free millimeter‐scale implantable neurostimulator for recording and outward transmission of neuroelectric signals. Reproduced with permission.^[^ ^279^ ^]^ Copyright 2022, Springer Nature. d) Schematic of a retinal stimulation prosthesis induced by ultrasound, which can be properly programmed and wirelessly transmitted via an external ultrasound transducer to an ultrasound field having a spatial acoustic power distribution. Reproduced under the terms of CC‐BY license.^[^ ^284^ ^]^ Copyright 2022, Laiming Jiang et al., published by Springer Nature.

Electrical stimulation has been widely studied and used to treat neurological disorders affecting the brain. Controlling the timing and intensity of the release of electrical signals can provide precise stimulation and modulate neural activity to a normal state. However, current implantable electrode systems require external battery power and typically need to be replaced every 3–5 years, thereby increasing the risk of infection with frequent procedures. Battery‐free electrical stimulation devices implemented using acoustic energy harvesters are well suited for overcoming this problem. An ultrasound energy‐harvesting device for deep brain stimulation was described by Zhang et al. (**Figure** **11**a).^[^ ^35^ ^]^ The device consists of a 6 × 6 array of Sm‐doped Pb (Mg_1/3_Nb_2/3_) O_3_‐PbTiO_3_ (Sm‐PMN‐PT) single crystals, which can generate a transient power density of 1.1 W cm^−2^ and an average charging power of 4270 ± 40 nW in vitro. Deep brain stimulation experiments in rats showed that the device could activate the periaqueductal gray matter (PAG) in the midbrain for analgesia. A neurostimulator based on a high‐performance miniature soft piezoelectric‐triacontrast‐nanogenerator (PTNG) was developed by Chen et al. (Figure 11b).^[^ ^277^ ^]^ applying programmable ultrasound pulses, PTNG could generate 0.9 mA of current and Parkinson's disease symptoms were relieved under stimulation.

The concept of a smaller neurostimulation unit (neurodust) developed using MEMS manufacturing technology was first proposed by Maharbiz et al. at the University of California, Berkeley.^[^ ^278^ ^]^ Attached to neurons at the corresponding sites, they are capable of neurostimulation by receiving external ultrasound, recording changes in neuroelectric signals, and sending them outward. More recently, the group has proposed a millimeter‐sized wireless “StimDust” stimulation motor that integrates piezoelectric ceramic sensors, energy storage capacitors, and integrated circuits (Figure 11c).^[^ ^279^ ^]^ A single link from a customized external transceiver to StimDust provided power and bidirectional communication, thus reducing its size. A customized time‐coded wireless ultrasound protocol dynamically sets the stimulation waveform, thereby significantly reducing the power consumption of the motes, while maintaining a high degree of timing control. By attaching hundreds or thousands of independent sensing nodes to nerves at different locations to form a neural recording system, the amount of neural data recorded can be significantly increased, providing a viable pathway for the realization of brain‐computer interfaces. Ning et al. proposed a wireless implantable neuroelectric stimulation (WINS) device based on a MEMS ultrasound scheme.^[^ ^280^ ^]^ It realizes the wireless connection of miniature electrodes through ultrasound, with a size of 5 mm × 5.5 mm × 2.5 mm, and is able to achieve an output power of 3.24 µW. The WINS was implanted onto the sciatic nerve of rats and electrically stimulated by applying ultrasound in vitro. Muscle tremors were observed, and muscle activities were recorded by EMG. Tang et al. developed an injectable and bioabsorbable intracranial monitoring sensor based on a metal gel.^[^ ^281^ ^]^ The sensor had a cubic size of 2 × 2 × 2 mm^3^. The metagel contains periodically arranged air columns that are slightly deformed due to changes in the physiological environment, resulting in a corresponding change in the acoustic emission spectrum.

Retinal prostheses developed for visual nerve stimulation using ultrasound principles are also an important research direction in neurostimulation.^[^ ^282^, ^283^ ^]^ The main components of such prostheses are an external detector and an implantable stimulation element. The visual information obtained by the external detector is transmitted to the implanted part via wired or wireless means to stimulate the optic nerve. Jiang et al. developed a flexible ultrasound‐induced retinal stimulation prosthesis (Figure 11d).^[^ ^284^ ^]^ The programmed ultrasound field was emitted from an external two‐dimensional ultrasound array. The ultrasonic signal is received by the piezoelectric array in the prosthesis and converted into electrical energy to initiate a nerve response by electrodes implanted at the retinal location. Electrical energy is then stimulated to initiate a neural response via electrodes implanted in the retina. The device can induce an electrical threshold of retinal response at low and safe ultrasound intensities (∼1–50 nC) and can support an operating frame rate of 120 Hz, which is far superior to that of existing commercially available visual prostheses. Nonetheless, the device requires further improvements in the acoustic–electric coupling efficiency to maintain high electrical output at lower sound pressure levels and a reduction in the size of the individual piezoelectric receiver unit to achieve higher resolution and larger field‐of‐view areas. Lu et al. developed a noninvasive ultrasound retinal prosthesis for simultaneous imaging and stimulation.^[^ ^285^ ^]^ A customized ultrasound 2D array produces programmed stimulation in the retina through simultaneous three‐dimensional (3D) imaging guidance and autofocus technology, which stimulates the brain's visual centers to recreate images. The prosthesis demonstrates a resolution close to that of PRIMA 24, used in clinical practice (less than 100 µm), and also provides a full‐size view of the retina, providing a more informative pattern.

Generating sound waves and wirelessly and noninvasively stimulating nerves using piezoelectric devices, especially focused ultrasound, is a promising approach. Focused ultrasound allows precise control of ultrasound focus and energy delivery and can safely modulate nerve centers with low energy transmission loss deep to deep nerve tissues.^[^ ^257^ ^]^ Various theories have been proposed to explain the mechanism of neuromodulation by ultrasound, including lipid bilayers and ion channels, mainly through mechanical action and acoustic cavitation phenomenon after the focusing of acoustic waves.^[^ ^254^ ^]^ Leinenga et al. used focused ultrasound to achieve the removal of the blood‐brain barrier, which leads to the removal of amyloid‐β, the protein that is the main cause of Alzheimer's disease.^[^ ^286^ ^]^ Avoiding cranial constraints could further enable low‐intensity ultrasound in wearable devices using lower‐frequency transcranial ultrasound or ultrasound stimulation at different angles past the cranial window, to act as much as possible at the site of brain lesions. Ali R. et al. found that the reduction of amyloid‐β in brains with the use of focused ultrasound to open the blood‐brain barrier in conjunction with conventional therapeutic means was significantly stronger than treatment without the assistance of focused ultrasound.^[^ ^287^ ^]^ In patients with mild Alzheimer's, focused ultrasound used in combination with aducanumab resulted in fewer abnormal responses. This stimulation can be achieved through mechanical, thermal, or acoustic cavitation produced by focused ultrasound waves in a specific area. A wireless wearable brain stimulation system was proposed by Kim et al.^[^ ^288^ ^]^ which customized PZT transducer customized PZT transducer produces an output ultrasound with a center frequency of 457 kHz, a peak pressure of 426 kPa, a weight of 20 g, and allows for a full range of motion. Stroke rehabilitation following temporal middle cerebral artery occlusion (tMCAO) in active rats demonstrated the ability of the device to produce both short‐ and long‐term results.

### 4.4 Acceleration of Wound Healing

**Figure 12:**
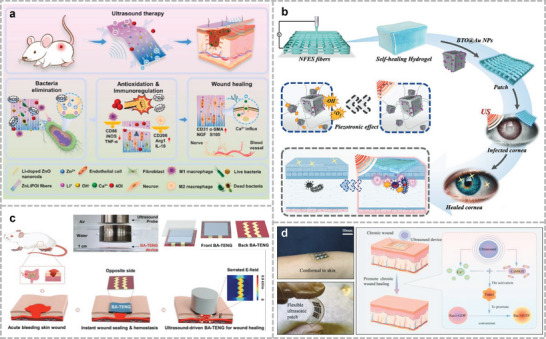
Ultrasound–assisted bandages and patch for wound therapy. a) Schematic of the therapeutic mechanism of novel electroactive composite device consisting of lithium‐doped ZnO/PLLA microfibres coated with antioxidant 4OI. Reproduced with permission.^[^ ^293^ ^]^ Copyright 2023, Elsevier. b) Schematic of composite patch treatment consisting of recombinant human collagen (RHC) hydrogel, near‐field electrospinning (NFES) microfibers, and gold nanoparticle‐decorated barium tetragonal titanate (BTO@Au) used for the treatment of keratitis, where ultrasound irradiation generates reactive oxygen species and thus destroys bacteria in the wound. Reproduced with permission.^[^ ^104^ ^]^ Copyright 2024, Wiley. c) Flexible ultrasound patch for the treatment of chronic wounds that accelerates wound healing by stimulating Rac1 in the dermis and epidermis with ultrasound signals. Reproduced with permission.^[^ ^36^ ^]^ Copyright 2021, Wiley. d) Bio‐adhesive triboelectric nanogenerators (BA‐TENG) for wound sealing and accelerated wound healing with ultrasound. Reproduced with permission.^[^ ^296^ ^]^ Copyright 2021, Wiley.

Ultrasound has many advantages as a therapeutic tool, including an associated high safety threshold, high stimulation intensity, and deeper penetration. They have been used in many therapeutic applications. Ultrasonic patches used for chronic wound healing have shown a significant value in clinical care. Wounds should be treated within the framework of tissue debridement, inflammation control, moisture balance, and epithelialization of the wound edge (TIME), with debridement being the key to treatment.^[^ ^289^ ^]^ Biofilms, which are structured bacterial communities found in more than half of all chronic wounds, are responsible for chronic inflammation.^[^ ^290^ ^]^ Recent studies have shown that ultrasound can effectively clean wounds and accelerate healing by increasing cellular activity, stimulating growth factor and protein synthesis, promoting fibrinolysis, and disrupting biofilm formation.^[^ ^291^, ^292^ ^]^ Therefore, flexible ultrasound transducers attached to wounds have significant application value. Wang et al. developed novel electroactive polymer devices using lithium‐doped ZnO/poly(L‐lactic acid) (PLLA) microfibers coated with 4 octyl itaconate (4OI) (**Figure** **12**a).^[^ ^293^ ^]^ During the whole wound repair, the device destroys bacteria in the wound through strong sonodynamic therapy, followed by anti‐inflammatory effects through the continuous release of 4OI and bioactive metal ions. Neurovascular efficacy was enhanced by mild electrical stimulation. Lithium doping not only improves the piezoelectric output but also enhances the generation of reactive oxide species (ROS), which effectively shortens wound healing time. Liu et al. developed a composite injectable hydrogel dressing with soft mechanical properties similar to extracellular matrix, significant bactericidal ability, and strong adhesion, as well as good self‐healing properties, and under US irradiation, the BT‐Gel showed excellent antimicrobial activity through rapid piezoelectric generation of ROS.^[^ ^294^ ^]^ In addition, the infected dermal defect model demonstrated significant efficacy in wound repair in terms of wound closure rate, granulation tissue regeneration, collagen distribution, inflammation reduction, and angiogenesis. Kong et al. presented an ultrasound‐responsive adhesive patch inspired by the cornea for the treatment of keratitis, using a recombinant human collagen (RHC) hydrogel, near‐field electrospinning (NFES) microfibers, and gold nanoparticle‐decorated barium tetragonal titanate (BTO@Au) compositions that showed excellent tissue adhesion (Figure 12b).^[^ ^104^ ^]^ NEFS not only reinforced the hydrogel but also stimulated the aligned growth of human corneal cells (HKs). Piezoelectric nanoparticles act as ultrasound sensitizers to convert acoustic energy into electrical energy and catalyze ROS production of reactive oxygen species. In vivo studies using a rat model of infected corneal wounds confirmed the superior efficacy of the patches.

In addition to removing wound bacteria to control inflammation, accelerating cell proliferation at the wound site is an effective method for scaling‐back wound healing. Low‐intensity ultrasound can remove inactivated tissues through microfluidic and cavitation effects, selectively emulsify dead and dying tissues, and stimulate the cell membranes of surrounding healthy cells. Lyu et al. designed a flexible ultrasound patch for the effective treatment of chronic wounds, which consisted of a flexible substrate, metal electrodes with an island‐bridge structure, piezoelectric ceramic cells, and flexible encapsulation layers (Figure 12c).^[^ ^36^ ^]^ Ultrasound excitation promotes the binding of intracellular Ca^2+^ to calmodulin‐dependent protein kinase II (CaMKII) and activates T‐lymphoma invasive metastasis‐inducing factor 1 (Tiam1), which accelerates the conversion of Rac1‐GTP to activate senescent or inactivated fibroblasts to accelerate the treatment of chronic wounds. Similarly, Shi et al.^[^ ^295^ ^]^ prepared flexible P(VDF‐TrFE)/BTO membranes capable of converting ultrasonic energy into electrical energy, and the piezoelectric properties of the BTO addition at 1.5 wt% were optimal for an ultrasonic stimulation at 1.0 W cm^−2^ with a voltage of 4.87 V and a current of 326 µA. The electric field promoted cell proliferation and migration, and the healing time of wounds with this device was five days shorter than that of the control group. Meng et al. developed a bioadhesive TENG (BA‐TENG) as first aid for rapid wound closure and hemostasis (Figure 12d).^[^ ^296^ ^]^ This device has faster and stronger adhesion speed and toughness. It is capable of generating a uniform electric field (0.86 kV/m driven by ultrasound, which can be used for accelerated wound healing. Wound‐healing experiments in mice demonstrated that the BA‐TENG had a faster healing speed.

### 4.5 Ultrasonic‐Assisted Drug Delivery

**Figure 13:**
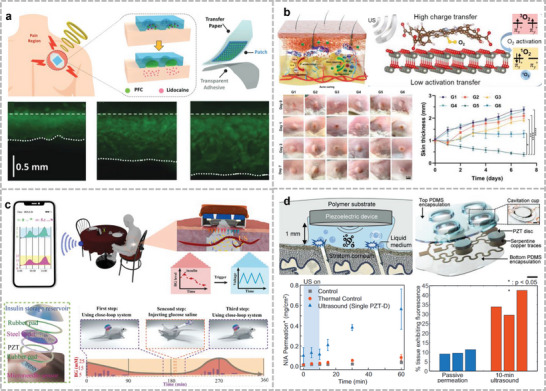
Ultrasound–assisted drug delivery. a) Non‐invasive topical drug delivery by acoustic droplet vaporization (ADV), which delivers the drug to the dermis and adjusts the amount of penetration by adjusting the applied ADV force. Reproduced with permission.^[^ ^297^ ^]^ Copyright 2018, Wiley. b) Schematic of acne treatment by ultrasound‐activated sodium hyaluronate needle patch and comparison of the results of different treatment strategies. Reproduced with permission.^[^ ^298^ ^]^ Copyright 2023, American Association for the Advancement of Science. c) Wearable, sensing‐controlled, ultrasound‐based microneedle smart system for diabetes management. Reproduced with permission.^[^ ^299^ ^]^ Copyright 2023, American Chemical Society. d) Conformal ultrasound patches for transdermal transport of nicotinamide by acoustic cavitation induced by intermediate frequency sonophoresis. Reproduced under the terms of the CC‐BY License.^[^ ^303^ ^]^ Copyright 2023, Chia‐Chen Yu, et al., published by Wiley.

Oral medication and injections are the two most common methods of drug ingestion, both of which have intrinsic limitations in clinical treatment. After oral administration, medicines are decomposed and absorbed by the digestive system, spread to the whole body through the circulatory system, and cure sick organs or tissues. The liver metabolizes most of the drug ingredients during this process. This process takes a longer time for the drug to work, and drugs that spread throughout the body are more likely to cause side effects. Although more of the drug is retained than taken orally, there is a risk of infection with the use of syringes, and the use of intravenous injections takes longer, which is inconvenient for patients. The use of microneedle patches with an ultrasound‐assisted release causes less skin damage and minimal pain. In addition, microneedle patches are simple and easy to administer, and can be self‐administered under the supervision of a physician with the ability to interrupt administration at any time in the event of discomfort. Soto et al. introduced a wearable transdermal patch that utilized the acoustic droplet vaporization (ADV) approach to achieve fast noninvasive drug delivery (**Figure** **13**a).^[^ ^297^ ^]^ ADV creates high localized stress to enhance permeability when a perfluorocarbon (PFC) emulsion is triggered and vaporized by ultrasonic stimulation. The degree of drug penetration was adjusted by regulating the magnitude of the local stress generated by the ADV. Xiang et al. developed sonocatalytic anti‐bacterial nanomaterials in microneedles to treat acne (Figure 13b).^[^ ^298^ ^]^ Ultrasonic field causes oxygen to gain more electrons and is converted into an excited state, which produces a large amount of reactive oxygen species and kills the bacteria. Under the stimulation of an ultrasonic field for 15 min, the patch killed 99.73% of P. acnes and cured the acne effectively, providing a highly effective and minimally invasive treatment option for patients with acne.

In addition to flexible drug microneedle patches that use external ultrasound, microneedles integrated with flexible miniature ultrasound transducer arrays can be used to modulate the rate of drug release by controlling the start/stop states of the transducer and related parameters. Luo et al. built a closed‐loop control microneedle system to stabilize glucose concentration (Figure 13c).^[^ ^299^ ^]^ Sensing microneedle detect changes in the glucose levels using an electrochemical approach. If the interstitial glucose concentration exceeds the threshold value, an ultrasonic insulin pump releases insulin through the delivery microneedle. Such wearable hypoglycemic management devices prevent patients with diabetes from forgetting periodic injections. Xu et al. integrated active acoustic meta‐materials into compact therapeutic devices.^[^ ^300^ ^]^ The localized ultrasound stream drives drugs to pass through the porous metamaterial structure into deep tissues to achieve rapid and on‐demand acute disease. Ashkan et al. constructed needle‐shaped microfabricated electrochemical probes to generate microbubbles.^[^ ^301^ ^]^ After the microneedle is inserted at the tumor site and a pulsed direct current is applied, excessive microbubbles are produced, inducing perforation of the cell membrane under ultrasound stimulation. This process enhances the absorption of anticancer drugs, which improves the efficacy of targeted cancer therapies.

Ultrasound can open the central nervous system barrier and blood‐brain barrier in a noninvasive manner and also acts as a trigger for drug release, interacting with microbubbles for noninvasive drug delivery. When microbubbles are exposed to ultrasound, their size oscillates, and they interact with nearby biofilms, thereby enhancing their permeability.^[^ ^302^ ^]^ Li et al. introduced a stretchable electronic facial‐mask platform to promote drug delivery.^[^ ^37^ ^]^ A piezoelectric array with a resonant frequency of 1 MHz was integrated on a face‐like encapsulation layer, and its function in drug delivery through surface skin was verified by animal skin experiments with the help of high‐frequency sonophoresis, which illustrates considerable medical potential. The conformal ultrasound patch developed by Yu et al. utilizes intermediate‐frequency sonophoresis (IFS) (100 kHz to 1 MHz) to induce acoustic cavitation, which can break down bubbles into smaller sizes and enhance the penetration dynamics for targeted delivery (Figure 13d).^[^ ^303^ ^]^ A 1 mm‐deep reservoir space provides the necessary space to induce inertial cavitation, convective mixing, and microjets. With further miniaturization of this device, it would be possible to replace oral and needle delivery methods.

## 5 Ultrasonic Diagnosis

Eight systems in the human body support the normal physiological activities of individuals, and each system has different structural characteristics. Examination of each body system can help doctors detect and diagnose diseases early, and effectively detect and evaluate abnormal signs. Compared to other detection techniques, ultrasound does not produce any radioactive radiation and is more suitable for special patients, such as pregnant women and children. This section summarizes and categorizes the flexible ultrasound transducers that have emerged in recent years for clinical aid diagnosis, according to their application scenarios.

### 5.1 Hemodynamics Measurement

Human blood signals are fundamental indicators of health and reflect life characteristics. Continuous monitoring of these signals is crucial for detecting potential cardiovascular diseases and providing timely treatment. Ultrasound can accurately detect changes in subepidermal arteries. However, currently available ultrasound probes do not fit well on the curved surfaces of the body and consume a lot of power, making them difficult to sustain. However, the flexible patches lack the penetration depth required to achieve accurate measurements.

**Figure 14:**
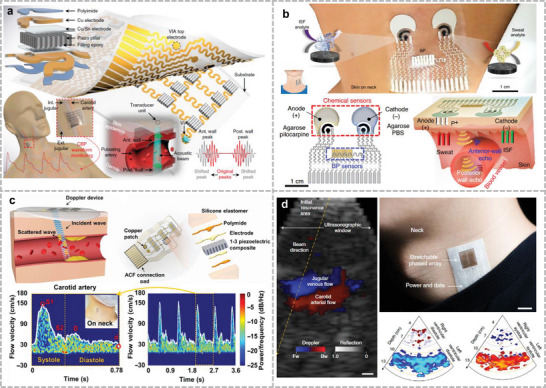
Hemodynamic monitoring by ultrasound methods. a) Schematic diagram of the structure of a wearable ultrasound transducer patch and the mechanism for monitoring arterial venous blood pressure waveforms. Reproduced with permission.^[^ ^304^ ^]^ Copyright 2018, Springer Nature. b) Schematic diagram of an epidermal patch integrating an ultrasound transducer and an electrochemical transducer for obtaining hemodynamic and sweat parameters in humans. Reproduced with permission.^[^ ^305^ ^]^ Copyright 2021, Springer Nature. c) A flexible continuous wave Doppler ultrasound device for real‐time, continuous monitoring of absolute blood flow velocity. Reproduced under the terms of CC‐BY license.^[^ ^29^ ^]^ Copyright 2021, Fengle Wang, et al., published by American Association for the Advancement of Science. d) Stretchable ultrasonic phased array for obtaining ventricular blood flow during cardiac diastole and systole by Doppler methods. Reproduced with permission.^[^ ^309^ ^]^ Copyright 2021, Springer Nature.

Ultrasound‐obtained vessel diameters included both ultrasound vessel wall tracking and ultrasound angiography. The artery diameter and blood pressure showed a positive correlation. Ultrasound is incident on the body through the time difference between the anterior and posterior wall echoes of the vessel to calculate the diameter of the blood vessel and determine the blood pressure. Wang et al. designed a flexible and stretchable ultrasound patch to integrate a transducer, flexible circuitry, and substrate using micro–nanomachining technology (**Figure** **14**a).^[^ ^304^ ^]^ This device developed strategic material integration and advanced micromachining technology to achieve state‐of‐the‐art functionality and suitable mechanical compliance, resulting in tight coupling with the human skin and continuous blood pressure monitoring at the center of the blood vessel. A comparison of the test results between a commercial sphygmomanometer and the patch demonstrated that the flexible patch can achieve accurate real‐time measurement of subcutaneous arteries and veins while circumventing measurement errors caused by joint movement, in addition to the patch's ability to combine with ECG to determine vascular sclerosis by calculating the cardiovascular pulse velocity (PWV). Sempionatto et al. developed a flexible patch with hybrid acoustic–electrochemical sensors (Figure 14b).^[^ ^305^ ^]^ The ultrasound transducer in the center of the patch is responsible for monitoring blood pressure and heart rate signals, whereas the left and right sides are positive electrodes and interstitial fluid (ISF) sensors, negative electrodes, and sweat biofluid sensors, respectively. The blood pressure transducer consists of eight piezoelectric transducers aligned with the carotid artery when used in the neck for optimal ultrasonic signaling. The optimal blood pressure signal can be selected from eight transducers, ensuring reliable sensing of blood pressure during motion in which the patch may be displaced. Li et al. went a step further in terms of wearable comfort by combining “S”‐shaped stretchable island bridge electrodes with fabric substrates.^[^ ^306^ ^]^ The detector can achieve an axial resolution of 330 µm superior to the Ecoflex‐based patch transducer and also shows good skin‐friendliness in breathability comparison experiments.

Doppler ultrasound technology has a wide range of applications in clinics and is used to examine blood vessels in various parts of the body. Using Doppler technology, it is possible to assess the blood flow rate of blood vessels, state of the vessel walls, and formation of blood clots, thereby helping to diagnose and monitor arteriosclerosis, thrombosis, thromboembolism, and other vascular diseases. However, Doppler modality measurements using conventional rigid probes require high handheld stability by the operator and may cause localized vascular compression. To solve this problem, Kenny et al. reported a hands‐free, continuous‐wave Doppler ultrasound patch for the rapid and easy detection of common carotid artery blood flow.^[^ ^307^ ^]^ When combined with an automated algorithm, the device tracks continuously recorded velocity waveforms and calculates hemodynamic indices, such as velocity‐time integrals. Implantable Doppler probes allow continuous monitoring, but are limited by wired connection requirements.^[^ ^308^ ^]^ Wang et al. developed a Doppler ultrasound patch for monitoring blood flow velocities in deeply buried arteries, which allowed real‐time continuous monitoring of absolute blood flow velocities without averaging (Figure 14c).^[^ ^29^ ^]^ The patch can obtain arterial blood flow velocities of at least 25 mm and present them in the form of a spectrum. With dual‐beam correction, the Doppler ultrasound patch avoids measurement errors introduced between the vessel orientation and skin angle, thereby improving the accuracy of absolute blood flow velocities. A large amount of blood velocity information is present in the systolic and diastolic activities of the heart, and the Doppler spectra of different cardiac cross sections can be used to assess the functioning of heart valves and the structural state of the heart. Wang et al. reported a skin‐conformable ultrasound phased‐array patch that can actively focus and steer the ultrasound beam within the range of −20° to 20° incidence angle and used the appropriate angle for interception to combine with the results of blood vessel size measurements to achieve real‐time cerebral blood flow (Figure 14d).^[^ ^309^ ^]^ The results of the vascular size measurement can be combined to realize a real‐time cerebral blood flow calculation. A conformal ultrasound patch for hands‐free volumetric imaging and continuous monitoring of cerebral blood flow was established by Zhou et al.^[^ ^27^ ^]^ The patch was attached at the cranial window location and used to reconstruct cerebral arterial structures using ultrafast ultrasound and volumetric Doppler images, and to monitor cerebral hemodynamic transformations in multiple scenarios. The echo amplitude can be further enhanced by using ultrasound contrast agents to achieve capillary‐level imaging. Krings et al. presented a dispersed liquid metal (LM) elastomeric composite with tunable acoustic properties that achieved an acoustic impedance of 4.8 MRayl and has low modulus (<1 MPa), and high stretch (>100% strain) for use as a matching layer in stretchable ultrasonic transducer arrays.^[^ ^33^ ^]^ Ultrasound devices that use this material as a matching layer can detect the movements of deep tissues and organ functions, such as heart valves and ventricular walls. Based on the current development of acoustic devices, the next generation of suitable ultrasound patches for blood parameter measurements should further enhance the degree of integration, such as by adding signal processing and wireless data transmission modules, as well as improving the penetration ability of flexible patches to enrich the richness of in vivo physiological information, so that the next generation of devices can be truly used in clinical and home healthcare (**Table** **4**).

**Table 4:** Wearable ultrasonic transducer for hemodynamics measurement.

Active material	Sensing mode	Resonant frequency	Penetrating depth	Refs.
PZT	ToF	1.3 MHz	2 cm	[310]
1–3 composite (4 × 5 array and phase array)	doppler	7.5 MHz/2 MHz	14 cm	[309]
1–3 composite (4 × 5 array)	ToF	7.5 MHz	4 cm	[304]
PMUT	ToF	161 kHz	–	[311]
1–3 composite (3 × 3 array)	doppler	5 MHz	2.5 cm	[29]
PMUT	ToF	5 MHz	4 cm	[312]
PZT	ToF	7 MHz	–	[305]

### 5.2 Continuous Organs Imaging

Ultrasonography is one of the most important methods for determining the condition of organs and tissues in the human body. Acoustic imaging methods can be used to obtain the structure of organs such as the liver and kidneys and to detect diseases such as stones and tumors. Although many wearable acoustic devices have been developed, these devices use a rigid ultrasound probe mechanically attached to the human skin and use the probe itself or an additional flexible transducer to obtain the echo signal, which can result in acoustic loss owing to the inability to fit the skin properly, affecting the quality of the image. Replacing rigid transducers with arrays of flexible ultrasound transducers allows image acquisition in curved body parts, improves image quality, and enables long‐term monitoring and treatment. However, arrayed imaging patches require algorithmic corrections because of unpredictable changes between units during stretching. A large spacing between cells must be maintained to avoid grating‐lobe artifacts. These issues combined to challenge the development of flexible acoustic imaging.

The human body causes changes in the imaging quality during movement, and maintaining a firm fit is the primary solution to this problem. Wang et al.^[^ ^313^ ^]^ combines the advantages of elastomers and hydrogels by encapsulating a soft hydrogel in a thin elastomeric membrane coated with a bioclastic material consisting of a chitosan‐polyacrylamide interpenetrating polymer network (10 wt%) and water (90 wt%). Using this mixture as a coupling agent enhanced the elasticity of the rigid transducer array, which maintained its adhesion for 48 h. The array also had a high density of piezoelectric cells (400 per square meter), enabling a minimum axial resolution of 0.25 mm.

**Figure 15:**
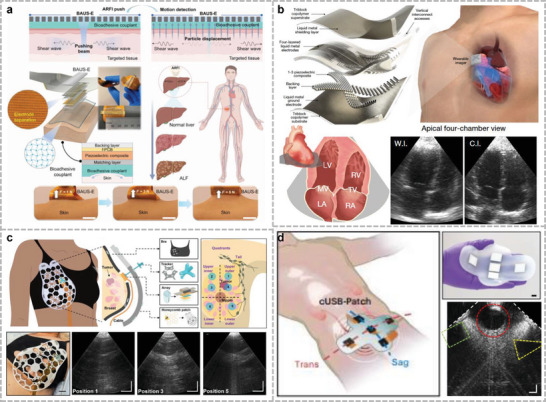
Continuous organ imaging with wearable ultrasonic transducer. a) Wearable bio‐adhesive ultrasound elastography (BAUS‐E) generates acoustic radiation force impulses (ARFI) to induce shear waves for elastography measurements, which can be used to observe stiffness changes in the human liver. Reproduced with permission.^[^ ^314^ ^]^ Copyright 2024, American Association for the Advancement of Science. b) Schematic of wearable ultrasound cardiac imager and ultrasound image of four ventricles. Reproduced under the terms of CC‐BY license.^[^ ^22^ ^]^ Copyright 2023, Hongjie Hu et al., published by Springer Nature. c) Conformable ultrasound breast patch (cUSBr‐Patch) to allow large area, deep scanning, and multi‐angle breast imaging. Reproduced with permission.^[^ ^315^ ^]^ Copyright 2023, American Association for the Advancement of Science. d) Conformable ultrasound bladder patch for bladder volume assessment. Reproduced with permission.^[^ ^30^ ^]^ Copyright 2024, Springer Nature.

The imaging patches described above can have a sufficiently comprehensive impact on most organs, such as the stomach and heart. However, organs with specific lesion symptoms require different acoustic modalities depending on the diagnostic needs; for example, changes in liver stiffness during the progression from fatty liver to cirrhosis can only be observed in the mid to late stages of the lesion by B‐mode ultrasound. Liu et al. developed wearable bioadhesive ultrasound elastography (BAUS‐E) that can continuously measure liver stiffness (**Figure** **15**a).^[^ ^314^ ^]^ The transducer array generates an acoustic radiation force impulse (ARFI) as an excitation source that produces shear waves for elastographic measurements. Further adherence to the skin by a bioadhesive hydrogel coupling agent and continuous wearable ultrasound elastography can be performed for 48 h. Changes in liver stiffness induced by d‐galactosamine (d‐Gal) in ALF rats were measured. Hu et al. developed an orthogonal flexible ultrasound imager using various anisotropic 1–3 piezoelectric composite compositions that can be used for human cardiac imaging and can continuously capture ventricular signals at different angles (Figure 15b).^[^ ^22^ ^]^ A deep learning algorithm that continuously extracts the left ventricular volume was developed to adapt to changes in the relative positions of the array elements owing to the curved contours of the human left thorax, thus obtaining key index parameters of the human heart.

The relatively small size of the flexible patch compared with most tissues or organs in the human body makes it difficult to image a single probe at a fixed location in a single session to visualize the organ structure. Therefore, it is feasible to use multiple probes and recover as much of the structure of an organ as possible using orthogonal images. Du et al. proposed a conformal ultrasound breast patch (cUSB‐Patch) consisting of a one‐dimensional phased array and an easy‐to‐manipulate nature‐like patch design, which provides large‐area, deep‐tissue scanning, and multi‐angle, reproducible breast imaging while avoiding the drawbacks of conventional ultrasound imaging techniques (Figure 15c).^[^ ^315^ ^]^ The patch has a maximum imaging depth of 80 mm and can achieve 0.25 mm axial resolution and 1 mm longitudinal resolution at 30 mm. Six 1–3 polymer phased arrays were mounted on an external patch with a honeycomb structure that fitted the curved surface of the chest. Each array can be individually rotated to capture thymic tissue sections from different angles, enabling the patch to aid in self‐screening for individuals at high risk of breast cancer and in the early detection of lesions through high‐frequency screening. Zhang et al. reported a conformable ultrasound bladder patch (conformable ultrasound bladder patch cUSB‐Patch) (Figure 15d).^[^ ^30^ ^]^ It is based on multiple‐phased arrays embedded in a stretchable substrate that can provide mechanical robustness, compliance, and in vivo volume organ monitoring. The central transducer reliably locates the initial central position of the bladder, and its five arrays allow imaging of the bladder from multiple angles, resulting in real‐time imaging and measurement without the need to move or rotate the transducer. With an axial resolution of 0.6 mm at a depth of 18.2 cm, and the same transverse resolution at a depth of −10.7 cm, it is suitable for imaging deep tissues and organs. The use of a stretchable substrate with a modulus of elasticity comparable to that of the human epidermis eliminates the need for uncomfortable ultrasound gel couplers.

### 5.3 Deep Tissue Monitoring In Vivo

**Figure 16:**
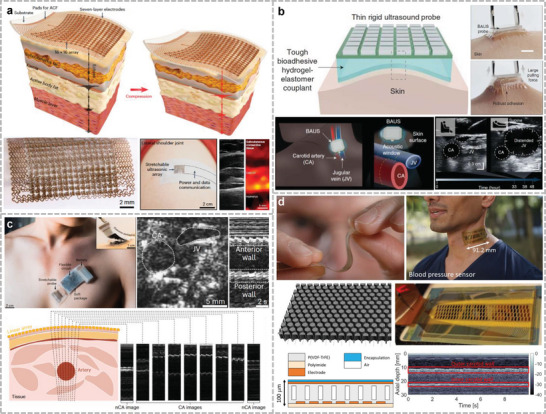
Ultrasonic imaging for deep tissue. a) Noninvasive elasticity measurements of tissues up to 4 cm subcutaneously by stretched ultrasound arrays and comparative validation with MRI. Reproduced with permission.^[^ ^316^ ^]^ Copyright 2023, Springer Nature. b) Simultaneous improvement of bio‐adhesion and ultrasound imaging quality with coupling agents made of soft, tough, anti‐dehydrating and bio‐adhesive hydrogel‐elastomer blends. Reproduced with permission.^[^ ^313^ ^]^ Copyright 2022, American Association for the Advancement of Science. c) Wearable ultrasound system for continuous imaging of human arteries and veins and machine learning methods to track moving targets. Reproduced with permission.^[^ ^318^ ^]^ Copyright 2023, Springer Nature. d) Flexible large‐area ultrasound array using column P (VDF‐TrFE) as acoustic transducer element for carotid artery imaging detection. Reproduced under the terms of CC‐BY license.^[^ ^319^ ^]^ Copyright 2024, Paul L. M. J. van Neer et al., published by Springer Nature.

Continuous monitoring of the biological properties of tissues can help physicians make early diagnoses, track and manage physiological conditions and tissue lesions, and assess the rehabilitation progress. The development of continuous imaging of deep human tissues differs from that of organs. The penetration depth and spatial resolution need to be further improved so as to enable the detection of subtle tissue lesions. Furthermore, owing to the varied locations of diseased tissues, wearable imagers for deep tissues should have the capability to image any part of the human body, presenting a considerable challenge to the conformability of the device. To address this issue, Hu et al. developed a low‐profile membrane‐based stretchable ultrasound probe to achieve accurate, artifact‐free, full‐field, and nondestructive detection on complex surfaces (**Figure** **16**a).^[^ ^316^ ^]^ Shear vibration is suppressed through the use of a high‐performance anisotropic type 1–3 piezoelectric composite material, crosstalk between the transducers is reduced, and longitudinal vibration is enhanced, thereby improving the overall sensitivity and signal‐to‐noise ratio The 10 × 10 array of addressable units can focus at different depths with a resolution comparable to that of a rigid probe. Image clarity and subsequent processing are crucial. The quality of coupling determines the degree of energy loss during the sending and receiving of acoustic waves in vivo, ensuring that the acoustic impedance is comparable to that of the human skin and has a high degree of adhesion and biocompatibility. Degradable implantable medical devices monitor and automatically degrade the body by being implanted into the patient's deep tissues for a specific treatment period. Liu et al. designed body‐absorbable conformal patches to monitor the homeostasis of biological tissues.^[^ ^46^ ^]^ The patch uses a hydrogel matrix that responds to pH changes. The matrix swells in the event of a pH imbalance and the bioresorbable metal indicator on the top changes its relative position. This change can be observed using a conventional ultrasound probe, which can be used to monitor postoperative leakage in the digestive system.

Ultrasound imaging of the arteries and veins beneath the epidermis and obtaining vessel diameters from the images provide clearer, more intuitive, and more accurate information about vessel diameters than vessel wall tracking methods. The diastolic and systolic velocities of the arteries can produce motion artifacts that lead to image distortion and blurring. Furthermore, the movements of the arteries themselves, along with the human body motion, can affect the position of the transducer relative to the blood vessel being examined. Such changes can result in an inaccurate depiction of the position of blood vessels in an image, which must be identified and addressed appropriately. Wang et al. reported a bioadhesive ultrasound device consisting of a thin, rigid ultrasound probe and a hydrogel‐elastomer mixture made of a soft, tough, dehydration‐resistant, and bioadhesive hydrogel‐elastomer mixture (Figure 16b).^[^ ^313^ ^]^ The device has a high‐performance piezoelectric element array density of up to 400 cm^−2^, ensures a constant relative position to the skin during physical activity, and uses a mixture that propagates ultrasound as far as possible into the body and remains adherent over a period of 48 h. The device successfully captured the cross and vertical sections of blood vessels at the neck in a variety of states of use, which can be used to estimate the diameter of the vessels and thus calculate real‐time blood pressure. To address the difficulty of maintaining bioadhesion, Ma et al. developed a controlled bioadhesion strategy based on the acoustic cavitation effect of ultrasound.^[^ ^317^ ^]^ The adhesion energy reached 100 J m^−2^ within 1 min and was stabilized within 10 min by the ultrasound acting on the anchors to penetrate the fixed tissues. Subsequently, the anchors were covered with a chitosan solution and a polyacrylamide‐alginate (PAAmalg) hydrogel and gelled. The experimental group treated with ultrasound showed a >15 times enhancement in the intrinsic work of adhesion in the fatigue fracture test compared to the control group without ultrasound treatment. Lin et al. designed miniature wearable patches that tracked physiological signals from tissues up to a depth of 164 mm (Figure 16c).^[^ ^318^ ^]^ Machine learning algorithms were used to classify the resulting continuous images of the tissue and select the optimal channel in real time, thus overcoming positional variations due to relative motion. In mobile subjects, the USoP can continuously monitor physiological signals for up to 12 h, including central blood pressure, heart rate, and cardiac output. Van Neer et al. used P(VDF‐TrFE) columns 40 µm wide and 80 µm high to form the PillarWaveTM ultrasound transducer, with an overall size of 9.1 × 1.4 cm^2^ and containing 2 × 64 elements (Figure 16d).^[^ ^319^ ^]^ The low acoustic impedance of the P(VDF‐TrFE) allows the device to have a broad bandwidth and a high axial resolution, thereby avoiding the necessity of a matching layer, resulting in a device thickness of only 100µm. Its mechanical flexibility was demonstrated by simple pulse‐echo measurements of the array wrapped around a 6 mm endoscope, and its potential for application was successfully applied to noninvasive imaging of the carotid artery to monitor arterial information.

### 5.4 Photoacoustic Imaging

The PA phenomenon was first reported by Bell in 1900. After light penetrates a certain depth of the tissue, the red blood cells flowing in the vessel absorb the energy of the photon transfer into heat energy and release it outside. When incident light is periodically modulated, the temperature of the target fluctuates, resulting in a periodic acoustic signal that can be detected by the surface transducer.^[^ ^320^ ^]^ This method had limited development until the emergence of laser technology, and many researchers developed numerous applications in vascular imaging. There are four main components: lasers, transducers, amplifiers, and data acquisition units. The performance of a photoacoustic imager is determined by the excitation wavelength and penetration depth of the laser, which affect the number of cells and molecules that can be detected and the imaging level of the blood vessels, respectively.

**Figure 17:**
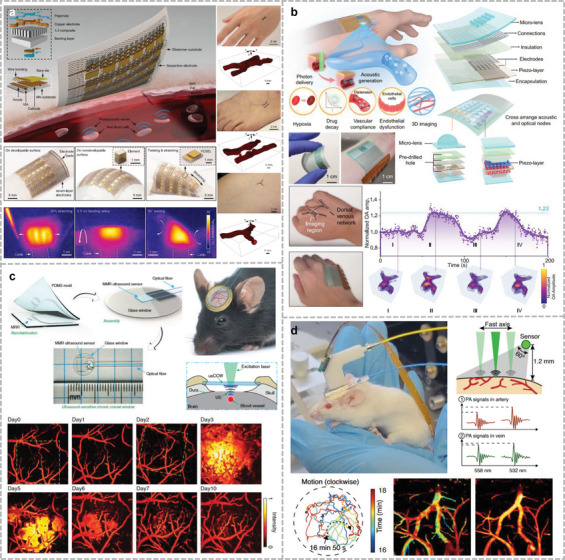
Wearable photoacoustic imaging. a) A flexible patch integrating an ultrasound piezoelectric unit and a vertical cavity surface emitting laser (VCSEL) allows the reconstruction of a three‐dimensional map of hemoglobin with sub‐millimeter resolution. Reproduced under the terms of CC‐BY license.^[^ ^45^ ^]^ Copyright 2022, Xiaoxiang Gao et al., published by Springer Nature. b) Flexible photoacoustic blood “stethoscope” for non‐invasive, multi‐parameter, and continuous cardiovascular monitoring with integrated micro‐focused lens array capable of generating a larger effective illumination area. Reproduced under the terms of CC‐BY license.^[^ ^321^ ^]^ Copyright 2023, Haoran Jin et al., published by Springer Nature. c) Ultrasonic sensing chronic cranial window (CCW) fabricated using soft nanoimprint lithography, transparent microwave resonator integrated into CCW to detect vascular structures in the brain. Reproduced under the terms of CC‐BY license.^[^ ^325^ ^]^ Copyright 2019, Hao Li et al., published by Springer Nature. d) Head‐mounted photoacoustic fiberscope for freely behaving mice. Reproduced under the terms of CC‐BY license.^[^ ^326^ ^]^ Copyright 2024, Xiaoxuan Zhong et al., published by Springer Nature.

Photoacoustic imaging patches formed by integrating a microlaser generator and an ultrasound transducer onto a flexible substrate can seamlessly fit into the skin and acquire deep tissue images.^[^ ^208^ ^]^ Gao et al. demonstrated a photoacoustic patch for three‐dimensional (3D) hemoglobin mapping of deep tissues (**Figure** **17**a).^[^ ^45^ ^]^ This photoacoustic patch integrates an array of ultrasonic transducers and vertical cavity surface‐emitting laser (VCSEL) diodes on a common soft substrate. The laser generated by the VCSEL can penetrate more than 2 cm and activate hemoglobin molecules to create acoustic waves. Transducers collect these signals and reconstruct 3D photoacoustic images that provide a more specific description of the artery and have significant implications for both research and clinical practice. Owing to the high operating power of a laser generator, when integrated with an ultrasonic transducer, there is a significant burden on the energy supply unit. The shallow penetration depth of the laser produced by the microlaser generator makes the solution of a laser produced by a high‐power laser through a focusing lens and fiber optics more widely used. Jin et al. report a flexible optoacoustic blood “stethoscope” (OBS) for noninvasive, multiparametric, and continuous cardiovascular monitoring, without requiring complicated procedures (Figure 17b).^[^ ^321^ ^]^ The OBS comprises a microlens array with predrilled holes for light delivery and an alternating piezoelectric sensor array as the acoustic receiver. They used a 5 × 5 PDMS‐fabricated convex microlens array as the optical focusing component to deliver light, and a piezoelectric PVDF film as the acoustic receiver. The array creates a relatively high light distribution uniformity compared to the center window and side illumination methods at the depth of the blind region, which is defined as the light intensity drop over 20 dB below the maximum. They verified its function through hypoxia, intravascular exogenous agent concentration decay experiments, and vascular occlusion tests on veins and arteries. The results proved that the relative percentage variation in the absolute optoacoustic amplitude can reflect hemodynamic parameters, providing a new perspective for predicting cardiovascular disease. Zhang et al. developed a wearable photoacoustic watch with an attached Pocket PC and a backpack with a laser and power supply for real‐time photoacoustic detection.^[^ ^322^ ^]^ The watch has a lateral resolution of 8.7 µm, a field‐of‐view (FOV) diameter of 3 mm, and a motorized adjustable focus to optimize the imaging plane for different individuals. In vivo imaging of blood vessels within the human wrist was performed under walking and cuff occlusion conditions to observe hemodynamic changes under different physiological states.

To further reduce the size and enhance the sensitivity of optoacoustic devices, some researchers have started to replace the vibration sensing component with optical interferometers such as Fabry‐Pérot, micro‐ring, and π‐phase‐shifted fiber Bragg gratings.^[^ ^323^ ^]^ Physical characteristics of ultrasound waves (e.g., intensity and frequency) affect the interference of laser resonators, and a read‐out system is adopted to convert them into voltage signals. Therefore, acoustic perturbations can be transformed into changes in optical properties with higher sensitivity than optoacoustic devices. In the Fabry‐Pérot interferometer, two flat mirrors are placed opposite to each other. When the relative positions of the mirrors were changed, the transmittance of the resonator for light with different wavelengths changed. Researchers have attempted to apply this interference model to the PAI. Ma et al. demonstrated an optically transparent microfibre ultrasound sensor with a needle‐shaped focus.^[^ ^324^ ^]^ A pair of high‐reflectivity fiber Bragg gratings (FBGs) clamp a ring‐shaped microfiber to form an ultrasound‐sensitive inline fiber‐optic Fabry‐Perot cavity. The ring structure increases the anti‐interference capability of other lights and the sensitivity to ultrasound waves. Li et al. developed a low‐cost soft nanoimprint lithography (sNIL) method to fabricate a disposable ultrasound‐sensing chronic cranial window (usCCW) comprising a transparent MRR‐based ultrasonic detector integrated into the inner surface of the CCW (Figure 17c).^[^ ^325^ ^]^ The MRR supports optical resonance (whispering gallery modes) with a reasonably high Q‐factor, which amplifies the ultrasound‐induced deformation of the waveguide into a frequency shift of the resonance modes in the optical spectrum. The MRR is encapsulated in an acoustic impedance‐matched protection layer to avoid scattering losses caused by contamination. usCCW allows unobstructed access to cortical areas while performing 3D isometric PAM imaging with a single capillary resolution. Zhong et al. developed a head‐mounted photoacoustic fibrescope for cerebral imaging in a freely behaving mouse (Figure 17d).^[^ ^326^ ^]^ The imaging probe uses two optical fibers for photoacoustic excitation and detection. A microelectromechanical system (MEMS) scanner was used for raster scanning of the laser beam, and the acoustic wave was detected using a fiber‐optic sensor with two orthogonally polarized lasers with slightly different lasing frequencies. The x‐ and y‐polarized laser beams have identical frequency shifts in the torsional‐radial mode vibration state. Consequently, the acoustically induced lasing frequency change can be measured in the radio frequency range.

## 6 Other Frontier Research

**Figure 18:**
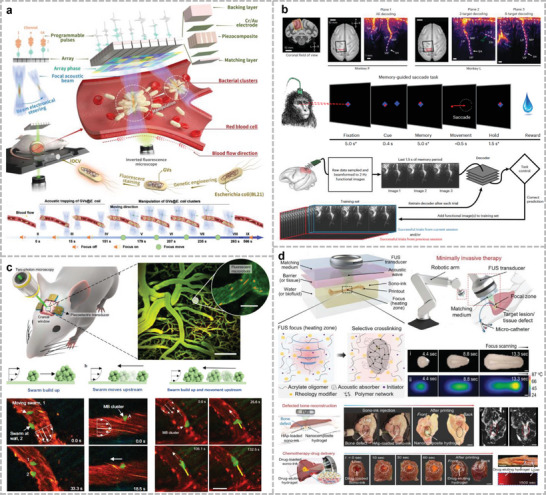
Other frontier research in acoustic field. a) Schematic of acoustic tweezers realized using bacteria capable of producing large numbers of submicron microbubbles. Reproduced under the terms of CC‐BY license.^[^ ^43^ ^]^ Copyright 2023, Ye Yang et al., published by Springer Nature. b) Schematic of closed‐loop ultrasound brain‐machine interface (BMI) system using focused ultrasound for use in rhesus monkeys. Reproduced under the terms of CC‐BY license.^[^ ^328^ ^]^ Copyright 2023, Whitney S. Griggs et al., published by Springer Nature. c) Autonomous intravascular aggregation and propulsion of microbubble‐containing microrobots using an ultrasound‐activated approach to vascularization. Reproduced under the terms of CC‐BY license.^[^ ^44^ ^]^ Copyright 2023, Alexia Del Campo Fonseca et al., published by Springer Nature. d) In vivo ultrasound 3D printing using focused ultrasound. Reproduced with permission.^[^ ^330^ ^]^ Copyright 2023, American Association for the Advancement of Science.

With the development of materials and medical technology, the applications of acoustic devices have gradually expanded to more sophisticated areas. The increased device power allows focused ultrasound to produce a direct mechanical action in the human body. It enables the stimulation of brain nerves for therapeutic purposes without the need for craniotomy and also allows for precise control on a cellular scale by using ultrasound, realizing the control and guidance of cells in the body. Wu et al. used gas particles (GV) for the first time in a genetically encoded ultrasound actuator that enhanced and altered the signs of acoustic radiation forces (ARFs) perceived by cells in ultrasound. GVs can be internalized by mammalian cells for selective activation and are subsequently degraded by the same cells, which provides a unique strategy for “no‐scalpel” labeling and cellular activation. Yang et al. genetically engineered Escherichia coli to produce nanoscale microbubbles (**Figure** **18**a).^[^ ^43^ ^]^ A phased array generates an ARF at a specific depth to capture bacteria. The modified bacteria were subjected to 63 times more ARF than the control group. It can be manipulated in the blood circulation by programmable pulses after systemic administration to move in the opposite direction or flow into predetermined blood vessels on demand. It can also significantly improve the migration of bacteria in tumors, slowing down their growth rate, which provides a method of target‐driven therapy for medical care. Hou et al. used nanobubbles as ultrasonic neurostimulation media.^[^ ^327^ ^]^ By delivering nanobubbles to specific locations in specific mice, ultrasound activated specific areas of the mouse motor cortex, which induced EMG signals. It individually activates one of the two nearby deep brain regions, which induces different behaviors. This highly directional and reproducible brain neurotherapy modality precisely activates deep brain neural tissues and achieves noninvasive neuromodulation.

An acoustic brain‐computer interface can be realized by using focused ultrasound to monitor real‐time neural activity in the brain through the skull and then combining the acquired acoustic images with human commands. Griggs et al. constructed a closed‐loop brain‐computer interface system using focused ultrasound (Figure 18b).^[^ ^328^ ^]^ Rhesus monkeys were specifically trained to perform eye and hand movements while focused ultrasound images of the posterior parietal cortex were detected. These images were used to train artificial intelligence algorithms to correlate brain and body activities. Focused ultrasound avoids damage to any invasive surgical procedure to build brain‐computer interfaces.

Precise manipulation of microstructures or systems by ultrasound fields helps achieve more accurate targeted drug release and precise treatment of diseased tissues. Microbubbles can also be used for the in vivo guidance of microbots, which can be actuated by embedding microbubbles in the structure of the microbots and directing ultrasound to the region where the microbubbles are located. Fonseca et al. developed microbots for cerebrovascular in vivo navigation consisting of lipid‐shelled microbubbles that can be irradiated by ultrasound waves for autonomous aggregation and propulsion (Figure 18c).^[^ ^44^ ^]^ Using a diameter of 1.1–1.4 µm and a resonant frequency of 490 kHz, the use of ultrasound with operating frequencies far apart ensures that the microbubbles do not oscillate strongly thus avoiding damage to the surrounding tissue. The microbots self‐assemble in the cerebral vasculature and perform countercurrent movements at speeds of up to 1.5 µm s^−1^, and can move against blood flow of about 10 mm s^−1^. However, the continued inability to simultaneously control microflow in multiple vessels and real‐time noninvasive observation of deep tissue regions remain insurmountable challenges. However, overcoming the surface forces that dominate at this scale remains a significant challenge. Zhang et al. developed the sonotransformer, which is a micromechanical structure with a series of soft‐hinge connections that can be activated acoustically.^[^ ^329^ ^]^ This structure achieved millisecond response times in an acoustic field. Hinges provide the mechanical action required to deform the entire structure by concentrating the acoustic vibration energy, allowing the structure to fold and return to its original shape when the acoustic field disappears.

Focused ultrasound in vivo can drive the gas microbubbles in vivo to produce mechanical effects. It can also be analogous to the function of lasers in 3D printing as a scanning tool to achieve in vivo 3D printing, compared to lasers through the thermal effect or photochemical reaction to cure the material. Ultrasonic printing through acoustic thermoacoustic polymerization has a deeper in vivo depth of penetration; however, the development of the acoustic polymerization effect at the same time and the development of the biocompatibility of the printing material. However, the development of printing materials with both acoustic polymerization effects and biocompatibility is a major limitation of this technology. Kuang et al. investigated phase‐transition viscoelastic sonicated inks (hereafter, sonoinks), which simultaneously exhibited deep acoustic penetration, low acoustic wave flow, and rapid acoustic–thermal‐induced polymerization of free radicals at the same time (Figure 18d).^[^ ^330^ ^]^ Combined with the corresponding focused ultrasound writing technique for deep‐penetration acoustic volumetric printing (DAVP), DAVP utilizes the acoustic–thermal effect of the focused ultrasound focal spot in viscoelastic sonoinks to achieve rapid solidification of the material without the need to build a platform for constructing a 3D structure. Focused ultrasound is capable of generating acoustic pressures of several tens of MPa at the focal position (64 mm) with the focusing zone having a radius of 0.3–0.7 mm, thus allowing the realization of high‐resolution printing accuracy, printing complex structures directly in vivo.

## 7 Summary and Outlook

In this review, we summarized the current research advances in wearable sensors that target human health via acoustic signals. Based on recent research results, they are categorized into four areas of application: physiological acoustic signal detection, ultrasound in vivo function and communication, ultrasound‐assisted therapy, and new forms of ultrasound medicine or human–computer interaction methods. Advances in materials, signals, and data‐processing technologies over the past decade have contributed to the evolution of wearable sensors from rigid and bulky to their current flexible, miniaturized, and low‐power forms. Depending on the application scenario, wearable devices have produced different forms of evolution and are able to fulfill an increasing number of health‐monitoring functions in complex human forms. We believe that future challenges in the development of wearable acoustic sensors may arise from three aspects: the biocompatibility and performance of materials, further miniaturization of sensors, and the establishment of human sensor networks.

The development of high‐performance biocompatible active materials is of the highest priority. The active material determines key parameters (such as the bandwidth, sensitivity, and maximum SPL) in the acoustic field. Wearable acoustic sensors must consider critical issues such as acoustic impedance matching, nontoxicity, and form fitting. For example, lead‐free KNN and BNT with good electrical properties and biocompatibility have been demonstrated and are gradually replacing lead‐based piezoelectric materials in various ultrasound scenarios. In addition, naturally occurring biomaterials have many biological advantages and exhibit considerable potential for various applications.

The integration and miniaturization of wearable acoustic sensor devices are key factors for achieving efficient, convenient, and widespread applications. Miniaturization ensures that the sensor device can fit lightly on the body without interfering with daily activities and comfort. For implantable ultrasound devices, smaller dimensions allow for attachment to more delicate body tissues, including capillaries and nerve fibers. This form allows the detection of physiological information in subtle parts of the body with low power consumption. This method can be applied to detect the recovery of specific parts of the body after surgery and automatically degrade in vivo after the recovery cycle.

The combination of acoustics with other cutting‐edge fields can introduce new forms of application to acoustic detection technology. Many acoustic signals collected by wearable sensors can be analyzed using artificial intelligence tools to delve deeper into human health information contained in the data. This analysis identifies routine health indicators and detects potential health problems and provides early warning and preventive measures. Additionally, a health sensor network composed of multiple sensors can continuously monitor multiple dimensions and different parts of the human body. This multi‐dimensional monitoring not only improves the accuracy of a single data point but also provides a more comprehensive and accurate health assessment by comprehensively analyzing multiple data sources. For example, the simultaneous monitoring of heart rate, respiratory rate, voice changes, and skin conductivity provides a more comprehensive understanding of the user's physiological and psychological states. When the system detects an abnormal signal, it can send an immediate alert to notify the user or medical professional for prompt intervention.

A good cost‐effectiveness ratio is necessary for the successful commercialization of all advanced technologies. The commercial viability of the latest wearable technologies is difficult to directly assess using existing evaluation criteria because of their different functional implementation methods (e.g., conventional versus friction electric microphones) or the novelty of their usage scenarios. Although some advanced materials may enhance performance, their high cost may limit their mass production. Material heterogeneity and compatibility can also affect the process difficulty. Overly complex microstructures increase the number of processing steps, which increases the technical difficulty and makes it difficult to ensure device consistency. Wearable devices should have high fatigue strength and chemical resistance to ensure a good user experience and market acceptance. Therefore, to ensure performance and reliability, wearable technologies with relatively simple structures that use more common materials have a better price/performance ratio and greater development potential.

In conclusion, wearable acoustic sensors have promising multidisciplinary research and innovation prospects. Their development will significantly drive innovation in healthcare and HMI and significantly impact the medical field. It is foreseeable that more flexible and convenient treatment options will emerge and drive personalized medicine to new heights, leading to transformative advancements in healthcare.

## Conflict of Interest

The authors declare no conflict of interest.
